# A systematic review of the factors influencing microbial colonization of the preterm infant gut

**DOI:** 10.1080/19490976.2021.1884514

**Published:** 2021-04-04

**Authors:** Miriam Aguilar-Lopez, Andrew M. Dinsmoor, Thao T. B. Ho, Sharon M. Donovan

**Affiliations:** a Division of Nutritional Sciences, University of Illinois at Urbana-Champaign, Urbana, USA; b Department of Pediatrics, Morsani College of Medicine, University of South Florida, Tampa, USA; c Department of Food Science and Human Nutrition, University of Illinois at Urbana-Champaign, Urbana, USA

**Keywords:** Preterm infant, gut microbiota, gut colonization, mode of delivery, antibiotics, human milk, dysbiosis

## Abstract

Prematurity coupled with the necessary clinical management of preterm (PT) infants introduces multiple factors that can interfere with microbial colonization. This study aimed to review the perinatal, physiological, pharmacological, dietary, and environmental factors associated with gut microbiota of PT infants. A total of 587 articles were retrieved from a search of multiple databases. Sixty studies were included in the review after removing duplicates and articles that did not meet the inclusion criteria. Review of this literature revealed that evidence converged on the effect of postnatal age, mode of delivery, use of antibiotics, and consumption of human milk in the composition of gut microbiota of PT infants. Less evidence was found for associations with race, sex, use of different fortifiers, macronutrients, and other medications. Future studies with rich metadata are needed to further explore the impact of the PT exposome on the development of the microbiota in this high-risk population.

## Introduction

The early postpartum period is a critically important time for establishing the gut microbiota. Studies in full-term infants have shown that the characteristics of gut microbial communities are determined by multiple factors, including postnatal age, mode of delivery, diet, antibiotic exposure,^1^ geographic location, and ethnicity.^2^ The type of feeding (breastfeeding or formula feeding) and the introduction to solids are the most influential in shaping composition and function of the gut microbiota in the first year of life.^3,4^ By 2- to 3-years of age, the gut microbiota resembles an adult-like microbiota composition.^5^ However, other factors, such as the exposure to antibiotics and hospitalization, can disrupt this trend.^3,5^

Preterm infants, born less than 37 weeks of gestation, experience many physiological, medical, dietary, and environmental challenges that can detrimentally affect their microbial colonization. The rates of PT birth by cesarean section (C-section) are around 31% worldwide,^6^ and 64% in the USA.^7^ These rates are higher than the prevalence of C-section delivery in full-term infants, which is about 21%.^8^ Given their prematurity and compromised health status, PT infants can remain hospitalized in the neonatal intensive care unit (NICU) for an extended period of time after birth. The total length of stay varies depending on the growth and development of each infant. Infants born at an earlier gestational age (GA) and with lower birth weight spend more time in the NICU.^9^ As part of their medical care, PT infants receive many medications that can influence the gut microbiota, particularly antibiotics. Another critical factor in the treatment of these infants is how they are fed. The goal of the dietary treatments is to optimize the infant’s growth by providing adequate calories, macronutrients, and micronutrients via parenteral or enteral routes.^10^ To achieve the nutritional goals, PT infants can be fed different types of milk and fortifiers during the course of their hospitalization.^11^ Taken together, these factors can profoundly influence the establishment of the gut microbiota of infants born preterm.

The way the microbiome develops in early life is critically important, as key mutualistic relationships exist between the host, bacterial communities, and their metabolites. Additionally, the microbiome shapes immune development,^12,13^ and is implicated in cognitive development.^14^ If this homeostasis is altered by external factors, a dysbiosis in the gut ecosystem can occur, with a greater presence and abundance of pathogenic bacteria.^15^ In PT infants, the gut microbial composition is often characterized as dysbiotic,^16^ with slower acquisition and an overall lower prevalence of beneficial bacteria.^17^ This dysbiosis appears to be associated with a higher risk of developing serious complications including sepsis, and necrotizing enterocolitis (NEC),^16,18,19^ which can have detrimental long-term effects on the infant’s health, including disruption in neurodevelopment. Previous systematic reviews have investigated how various factors influence PT infants microbiome, including antibiotic use,^20^ enteral feeding,^21^ and the hospital environment.^22^ However, these factors do not work in isolation, and no previous systematic review has attempted to capture the full complexity of factors shaping PT infants microbiome. Thus, the goal of this review was to review the literature available regarding the impact of perinatal, physiological, pharmacological, dietary, and environmental factors on the composition of the gut microbiota of PT infants. By holistically examining the multifactorial influences on colonization of PT infant’s gut, gaps in the literature will be identified, which will highlight the opportunities for novel interventions aiming to optimize the establishment of these bacterial communities of infants born preterm.

## Methods

This systematic review was registered in the PROSPERO database (CRD42020131964) and was conducted according to the guidelines of the Preferred Reporting Items for Systematic Review and Meta-Analysis Protocols (PRISMA).^23^

### Data sources and search strategy

A systematic search was performed in four databases (PubMed/MEDLINE, Scopus, Web of Science, and the Cochrane Library) from May to July of 2019. The search terms included: “preterm infant”, “premature infant”, “extremely premature infant” “gut microbiome”, “gut microbiota”, “gastrointestinal microbiome”, “fecal microbiota”, “maternal health”, “gestational age”, “mode of delivery”, “C-section”, “cesarean section”, “immaturity”, “neonatal intensive care unit”, “NICU”, “hospital environment”, “hospitals”, “antibiotics”, “anti-bacterial agents”, “medication”, “parenteral nutrition”, “enteral nutrition”, “breastfeeding”, “human milk”, “mother’s milk”, “donor human milk”, “preterm formula”, “infant formula”, “probiotic”, “probiotics”, “prebiotic”, “prebiotics”, and “milk fortifier”.

### Study selection

To be eligible, studies needed to be focused on the gut microbiota of PT infants, conducted in human subjects, and be a cross-sectional, longitudinal, or s clinical trial study. Articles were excluded if they were not in English, no full-text was available, and were published before 2009, when advanced sequencing technologies were not widely used.^24^ After the literature search, all obtained articles were independently assessed by the two authors (MAL and AMD) to determine those to be included in the review. In the case of disagreements, a third author (SMD) resolved the discrepancies.

### Data extraction

The information extracted from each study included: author, year of publication, geographic location of the study sample, study design, sample size, length of study, intervention or exposure, (if applicable), intervention characteristics (if applicable), control group (if applicable), gut microbiota assessment method, 16S rRNA variable region (if applicable), sequencing platform (if applicable), alpha diversity, beta diversity, taxonomy, other gut microbiota related outcomes, and clinical outcomes.

### Quality of the evidence and risk of bias assessment

Eligible clinical trials were assessed using the Cochrane Collaboration Tool for assessing risk of bias (RoB2).^25^ This tool assesses potential research biases in five domains: bias arising from the randomization process, bias due to deviations from intended interventions, bias due to missing outcome data, bias in measurement of the outcomes, and bias in selection of the reported result. From these domains, an overall risk of bias was assigned to each study. A study was considered as “low risk of bias” if it showed low risk an all five domains, “some concerns” if it raised concerns in at least one of the domains, and “high risk of bias” if a study was high risk of bias in at least one of the domains, or scored “some concerns” in more than one domain.^25^ Cross-sectional or longitudinal studies were assessed using the Newcastle-Ottawa Scale (NOS) for observational studies.^26^ This tool measures four domains, including participant selection, comparability, exposure, and outcome. The scoring is based on number of stars, cross-sectional studies could receive up to six stars, and longitudinal studies could score a maximum of nine stars.^26^ All the selected articles were assessed by MAL and AMD.

## Results

### Study selection

**Figure 1.**
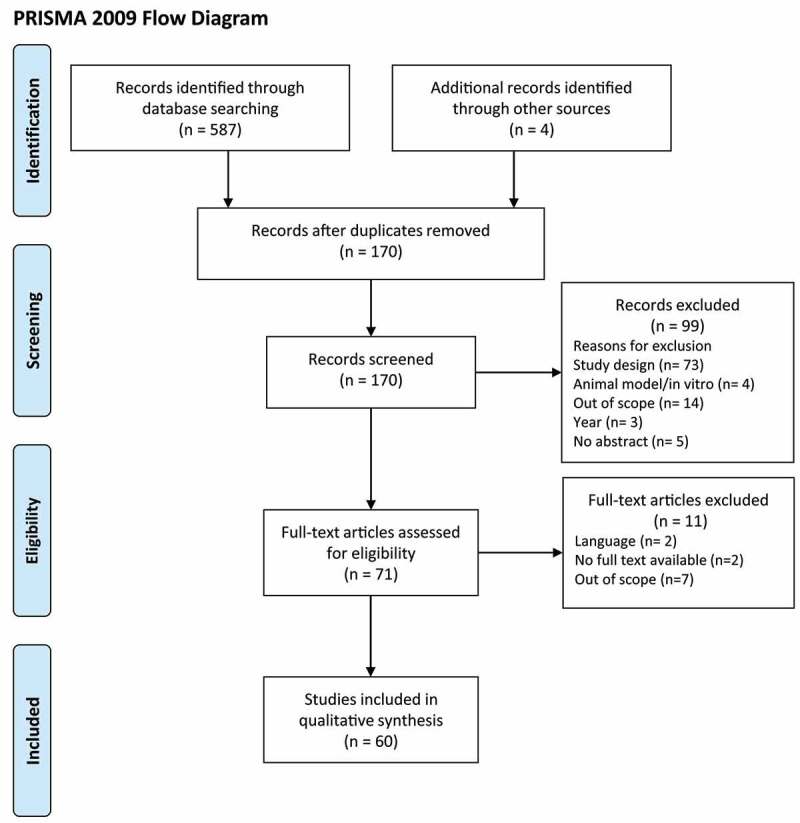
PRISMA flow diagram of search strategy

A total of 587 articles were identified through the database search, and four articles were retrieved through cross-reference. After removing duplicates, 170 articles were initially screened by title and abstract. At this step, 99 articles were excluded based on the study design (n = 73), studies performed in animal models or *in vitro* (n = 4), scope of the study (n = 14), year of publication (n = 3), and no abstract availability (n = 5). In total, 71 articles underwent full-text review. In this step, 11 articles were removed due to text not being available in English (n = 2), no availability of full text (n = 2), or scope of the study (n = 7). As shown in (Figure 1), a total of 60 articles were included in the qualitative synthesis.

### Study characteristics

Characteristics of the 60 articles are presented in (Table 1). The average sample if the included studies was of 50 infants. Twenty five percent of the studies were clinical trials (n = 15), and 75% observational studies (n = 45). The most common treatments from the intervention studies were the supplementation of prebiotics or probiotics (n = 14). The determination methods of the gut microbiota of PT infants, summarized in **Supplementary Table 1**, included bacterial DNA sequencing, bacterial culture, denaturing, and temperature gradient gel electrophoresis (DGGE and TGGE), terminal restriction fragment length polymorphism (T-RFLP), pulsed-field gel electrophoresis (PFGE), fluorescent *in situ* hybridization (FISH), matrix assisted laser desorption/ionization time-of-flight mass spectrometry (MALDI-TOF) and microarrays. Most studies used next-generation sequencing (NGS) technologies targeting the V3-V4, V4, and V3-V5 regions of the 16S rRNA bacterial gene. The platforms used included Ion Torrent, Roche 454 GS FLX Titanium, and Illumina technologies.

**Table 1.** Characteristics of studies included in the systematic review

Author	Year	Country	Study Design	Sample Size	Sample Characteristics	Intervention or Exposure	Length of Study
Adbulkadir, et al. ^27^	2016	USA	Clinical Trial	10	<32 weeks GA	Infloran®	Introduction enteral feeds to 34 weeks cGA
Aly, et al. ^82^	2017	Egypt	Clinical Trial	40	≤34 weeks GA	Unprocessed clover honey	d1 to d14 postnatal age
Arboleya, et al. ^65^	2015	Spain	Observational	27	24–32 weeks GA		d1 to d90 postnatal age
Armanian, et al ^83^	2016	Iran	Clinical Trial	50	<37 weeks GA ≤1500 g BW	GOS and FOS	d3 postnatal age until infants reached 150 ml/kg/day milk
Biagi, et al. ^68^	2018	Italy	Observational	16	32–37 weeks GA		d1 to d30 postnatal age
Brooks, et al. ^89^	2014	USA	Observational	2*	<37 weeks GA		d1 to d30 postnatal age
Brooks, et al. ^90^	2017	USA	Observational	50	<31 weeks GA <1250 g BW		d5 to d28 postnatal age†
Brown, et al. ^56^	2018	USA	Observational	35	<37 weeks GA		d1 to d90 postnatal age
Butcher, et al. ^37^	2017	Canada	Observational	54	<37 weeks GA <1500 g BW		d1 to d49 postnatal age
Cai, et al. ^46^	2019	Canada	Observational	20	<37 weeks GA <1500 g BW		d1 postnatal age to 4 weeks after introduction of enteral feeds
Chernikova, et al. ^32^	2016	USA	Observational	9	24–29 weeks GA		d1 to d54 postnatal age†
Chernikova, et al. ^50^	2018	USA	Observational	30	<37 weeks GA		Birth until discharge
Cong, et al. ^33^	2017	USA	Observational	38	28–32 weeks GA		d1 to d30 postnatal age
Costello, et al. ^62^	2013	USA	Observational	6	<37 weeks GA		d8 to d21 postnatal age
Dahl, et al. ^59^	2018	Norway	Observational	160	<37 weeks GA		d10 to 1-year postnatal age
Esaiassen, et al. ^53^	2018	Norway	Observational	66	<32 weeks GA	Infloran®	d1 to d120 postnatal age
Forsgren, et al. ^17^	2016	Finland	Observational	43	32–37 weeks GA		d14 to d180 postnatal age
Gibson, et al. ^60^	2016	USA	Observational	84	<33 weeks GA		48 h before and 48 after antibiotic exposure
Gómez, et al. ^61^	2017	Spain	Observational	16	≤32 weeks GA ≤1200 g BW		d1 to d21 postnatal age Second screening at 2-years postnatal age
Gregory, et al. ^51^	2015	USA	Observational	29	<32 weeks GA		d1 to d42 postnatal age
Gregory, et al. ^57^	2016	USA	Observational	30	<32 weeks GA		d1 to d42 postnatal age
Grier, et al. ^64^	2017	USA	Observational	95	23–37 weeks GA		Birth until discharge, second screening at 1-month and 1-year adjusted age
Gupta, et al. ^47^	2012	USA	Observational	76	≤34 weeks GA ≤1500 g BW	Histamine 2 receptor blockers	One time point at d62 postnatal age
Ho, et al. ^35^	2018	USA	Observational	45	<1500 g BW		d1 to d28 postnatal age
Ishizeki, et al. ^84^	2013	Japan	Clinical Trial	40	<37 weeks GA	*Bifidobacterium breve* or combination of *B. breve + Bifidobacterium. longum* subsp. *Infantis* + *B.longum* subsp. *longum*	Initiation of enteral feeds to 8 weeks after
Korpela, et al. ^38^	2018	Norway	Observational	50	<37 weeks GA ≤1500 g BW		d1 to d60 postnatal age
La Rosa, et al. ^48^	2014	USA	Observational	58	<37 weeks GA ≤1500 g BW		d1 to d30 postnatal age
Mai, et al. ^63^	2013	USA	Observational	28	≤32 weeks GA	PT infants with LOS and Healthy Controls	Birth until discharge
Millar, et al. ^73^	2017	UK	Clinical trial	115	<31 weeks GA	*B. breve*	Birth until 36 weeks cGA
Moles, et al. ^66^	2013	Spain	Observational	14	≤32 weeks GA ≤1200 g BW		Birth until discharge
Moles, et al. ^74^	2015	Spain	Observational	26	≤32 weeks GA ≤1200 g BW		Birth until discharge, second screening at 2-years postnatal age
Mshvildadze, et al. ^77^	2010	USA	Observational	27	<32 weeks GA		Birth until discharge
Normann, et al. ^67^	2012	Sweden	Observational	95	<28 weeks GA	PT infants with NEC and Healthy controls	d1 to d49 postnatal age
Parra-Llorca, et al. ^78^	2018	Spain	Observational	69	≤32 weeks GA ≤1500 g BW		One time point when full enteral feeds achieved
Pärtty, et al. ^85^	2013	Finland	Clinical Trial	34	32–37 weeks GA >1500 g BW	Polydextrose plus GOS or *Lactobacillus rhamnosus GG*	d30 to d365 postnatal age
Patel, et al. ^43^	2016	USA	Observational	12	<35 weeks GA <2000 g		d1 to d30 postnatal age
Poroyko, et al. ^79^	2011	USA	Observational	11	<37 weeks GA	Breastmilk or PT formula	One time point at 34–36 weeks cGA
Ravi, et al. ^54^	2017	USA	Observational	52	<37 weeks GA	PT infants with NEC and Healthy controls	d1 to d46 postnatal age†
Rougé, et al. ^86^	2009	France	Clinical Trial	94	<32 weeks GA <1500 g BW	*B. longum* BB536 and *L. rhamnosus* GG	Beginning of enteral feeds until discharge
Rozé, et al. ^88^	2017	France	Observational	94	<32 weeks GA		Birth until discharge
Sherman, et al. ^31^	2016	USA	Clinical Trial	120	<37 weeks GA ≤1250 g BW	Talactoferrin	d1 to d28 postnatal age
Sim, et al. ^52^	2014	UK	Observational	369	<32 weeks GA	PT infants with NEC and Healthy controls	d1 to d30 postnatal age
Soeorg, et al. ^80^	2017	Estonia	Observational	49	<37 weeks GA		d1 to d30 postnatal age
Stewart, et al. ^40^	2017	UK	Observational	46	<37 weeks GA	Infloran®	d1 to d100 postnatal age
Tauchi, et al. ^55^	2019	Japan	Observational	17	<37 weeks GA		From day 5 to 1 month of life
Underwood, et al. ^87^	2009	USA	Clinical Trial	90	<35 weeks GA	Culturelle® or ProBioPlus DDS®	d1 to d28 postnatal age or discharge
Underwood, et al. ^30^	2013	USA	Clinical Trial	21	<33 weeks GA <1500 g BW	*B. longum* subsp. *infantis* or *Bifidobacterium animalis* subsp. *lactis*	d1 to d35 postnatal age
Underwood, et al. ^28^	2014	USA	Clinical Trial	39	<33 weeks GA <1500 g BW	PT formula + GOS, or PT formula + HMF, or MOM + HMF, or MOM + BMF	For 5 weeks after initiation of enteral feeds
Underwood, et al. ^81^	2015	USA	Observational	14	<37 weeks GA	MOM	One time point at 30 weeks cGA
Underwood, et al. ^29^	2017	Australia	Clinical Trial	29	<37 weeks GA	*B. breve* M16-V	Initiation of enteral feeds to 3 weeks after
Wandro, et al. ^41^	2018	USA	Observational	32	≤1250 g BW		d1 to d75 postnatal age^1^
Westerbeek, et al. ^69^	2012	Netherlands	Clinical Trial	113	≤32 weeks GA ≤1500 g BW	GOS + FOS + AOS	d3 to d30 postnatal age
Younge, et al. ^76^	2017	USA	Clinical Trial	32	<37 weeks GA	Fish oil + Safflower	Initiation of enteral feeds to 10 weeks after
Younge, et al. ^58^	2019	USA	Observational	60	<28 weeks GA		Birth until 40 weeks cGA or discharge
Zeber-Lubecka, et al. ^44^	2016	Poland	Clinical Trial	55	25–33 weeks GA	Dierol®	d1 to d42 postnatal age
Zhou, et al. ^42^	2015	USA	Observational	38	<32 weeks GA	PT infants with NEC and Healthy controls	d1 to d60 postnatal age or discharge
Zhu, et al. ^70^	2017	China	Observational	36	28–37 weeks GA	Postnatal antibiotics	d1 to d7 postnatal age
Zou, et al. ^71^	2018	China	Observational	28	<32 weeks GA	Prenatal antibiotics	d1 to d60 postnatal age or discharge
Zwittink, et al. ^45^	2017	Netherlands	Observational	10	25–30 weeks GA		d1 to d42 postnatal age
Zwittink, et al. ^34^	2018	Netherlands	Observational	15	32–37 weeks GA	Postnatal antibiotics	d1 to d42 postnatal age

* Multiple sampling of the same infants throughout time, a total of 93 stool samples were collected.

† Follow-up varied among participants.

Infloran®: *Lactobacillus acidophilus* + *Bifidobacterium bifidum*; ProBioPlus DDS®: *Lactobacillus acidophilus* + *Bifidobacterium longum* + *Bifidobacterium bifidum* + *Bifidobacterium infantis* + inulin; Culturelle®: *Lactobacillus rhamnosus* GG + inulin; Dierol®: *Saccharomyces. Boulardii.*

AOS: acidic oligosaccharides; BMF: bovine milk-based fortifier; BW: birth weight; cGA: corrected gestational age; FOS: fructooligosaccharides; GA: gestational age; GOS: galactooligosaccharides; HMF: human milk-based fortifier; HMOs: human milk oligosaccharides; LOS: late onset sepsis; MOM: mother’s own milk; NEC: necrotizing enterocolitis; PT: preterm.

### Quality of the evidence

The RoB2 tool, applied to clinical trials, showed that 4/15 studies (26.7%) scored “Some concerns” for the risk of bias. The primary source of bias, summarized in **Supplementary Table 2**, came from the randomization process,^27^ deviation from the intended intervention,^28–30^ and the measurement of the outcomes.^31^ One study,^31^ was found to have high risk of bias, and thus it was not included in the description of the results. Evaluation of cohort and case-control studies using NOS are shown in **Supplementary Table 3** and **Supplementary Table 4**, respectively. Among the cohort studies, 39.4% had a score of nine stars (highest score possible. A score of six was obtained in 20 of the 33 cohort studies, because these studies consisted in only one group of PT infants. Therefore, these cohort studies had no score for the “selection of the non-exposed cohort” and “comparability of cohort on the bases of the design or analysis” sections. Lastly, all the case-control studies had an overall score of nine stars.

### Factors affecting the gut microbiota of preterm infants

#### Perinatal factors

##### Pregnancy complications

Four observational studies, shown in (Table 2), reported the effect of premature rupture of membranes (PROM),^32–34^ chorioamnionitis, which is the bacterial infection of the membranes of the placenta and amniotic fluid,^32,35^ prenatal antibiotics,^32^ and antenatal steroids^35^ on the gut microbiota composition of PT infants. Infants from mothers who had PROM and/or chorioamnionitis (diagnosed and confirmed by placental pathology) during pregnancy had lower alpha diversity over time compared to those infants whose mothers did not develop these complications.^32^ However, this association was significantly confounded by the use of antibiotics. Cong et al. found that PROM explained ~2% of the variation of the beta diversity from gut microbiota of PT infants.^33^ Chernikova et al. described that, regardless of the use of antibiotics, PT infants exposed to prolonged PROM had higher abundances of *Staphylococcus* and *Streptococcus* across time; these infants also showed faster increase in the abundance of *Enterobacter*, and lower colonization with *Clostridium* over time.^32^ In contrast, Zwittink et al. found no association between the gut microbiota composition of PT infants and the exposure to PROM.^34^ Infants exposed to chorioamnionitis during gestation, had greater abundances of *Serratia, Parabacteroides*, and *Bradyrhizobium* independent of the use of antibiotics.^32^ It is important to mention that *Bradyrhizobium* has been described as a common contaminant from NGS techniques which can be detected in samples with low microbial biomass.^36^ Another observational study found that the relative abundance of Gammaproteobacteria was positively associated with antenatal steroids.^35^ This same study demonstrated that PT infants showed two different gut bacterial community patterns described as clusters. Cluster 1 with low abundances of Gammaproteobacteria and Cluster 2 with high abundances of Gammaproteobacteria.^35^ When PT infants from Cluster 2 were exposed to chorioamnionitis (diagnosed by clinical sings) during gestation, the abundances of Gammaproteobacteria were lower, whereas PT infants from Cluster 2 exposed to antenatal steroids had higher abundances of Gammaproteobacteria.^35^

**Table 2.** Perinatal factors and gut microbiota composition of PT infants

Factor	Ref	Alpha diversity	Beta diversity	Taxonomy
***Pregnancy Complications***	^32^	↓ diversity across time in PT infants exposed to PPPROM and/or chorioamnionitis		↑ *Staphylococcus* and *Streptococcus* across time, faster increase of *Enterobacter*, and lower increase in *Clostridium* when exposed to PPPROM ↑ *Serratia, Parabacteroides*, and *Bradyrhizobium* when exposed to chorioamnionitis
	^33^	No association between PROM and Gini-Simpson diversity index	PROM explained ~2% of the variance from Bray-Curtis dissimilarity index	
	^35^			↓ Gammaproteobacteria when exposed to chorioamnionitis only in PT infants belonging to Cluster 2* ↑ Gammaproteobacteria associated with antenatal steroids only in PT infants belonging to Cluster 2*
	^34^			No association between PROM and gut microbiota composition
***Mode of Delivery***	^65^			↑ *Bacteroides* in vaginally delivered PT infants at 10 days postnatal age
	^37^	Over time, no differences in Shannon diversity index by mode of delivery in PT infants fed MOM	No association in between mode of delivery and Bray-Curtis dissimilarity index Mode of delivery explained ~1% of the variation in PT infants fed MOM	↑ Bacilli in PT infants fed MOM born via C-section during the first 3-weeks of postnatal age
	^46^		During late stage of enteral feeds† mode of delivery was associated with Unweighted UniFrac distances	
	^32^	↑ Simpson diversity index in PT infants born via C-section		↓ *Enterobacter, Pantoea, Citrobacter, Kluyvera, Erwinia* and *Klebsiella* in vaginally delivered PT infants
	^50^			↑ *Bacteroides* positively associated with vaginal birth
	^53^			At 7 days postnatal age, no differences in microbial composition by mode of delivery
	^51^			↑ *Bacteroides* over time in vaginally delivered PT infants
	^47^			↓ Proteobacteria in vaginally delivered PT infants
	^35^			↑ Firmicutes in PT infants born by C-section ↑ Gammaproteobacteria in vaginally delivered PT infants at ≤2 weeks postnatal age
	^38^	No association between observed OTUs and mode of delivery		↑ *Staphylococcus* in vaginally delivered PT infants No differences in *Enterococcus* and *Bifidobacterium* by mode of delivery
	^48^			Infants born <26 weeks GA via C-section: ↑ Bacilli and ↓ Gammaproteobacteria Infants born 26–28 weeks GA via C-section: ↑ Bacilli
	^77^	In meconium, and stool of >7 days postnatal age, no difference in Simpson diversity index by mode of delivery		
	^43^		No association between mode of delivery and Unweighted UniFrac distances	
	^54^			No association with microbial composition and mode of delivery
	^52^			↑ *Enterobacteriaceae* and ↓ *Clostridium* in vaginally delivered PT infants
	^40^	No differences in Observed OTUs by mode of delivery Vaginally delivered infants kept more OTUs from birth than C-section at 2-months postnatal age and after discharge	No association between mode of delivery and Unweighted UniFrac distances	During first week postnatal age, vaginally delivered PT infants belonged to cluster dominated by *Escherichia*, and PT infants delivered via C-section belonged to cluster dominated by *Klebsiella*
	^55^			No association with microbial composition and mode of delivery
	^41^	No differences in Shannon diversity index by mode of delivery	Mode of delivery explained 12% of the variation of Weighted UniFrac distances	Only vaginally delivered PT infants were colonized with *Bacteroides*
	^44^		No association between mode of delivery and PCA	After supplementation with probiotics‡, *Bacteroides* and *Parabacteroides* were only present in vaginally delivered PT infants Mode of delivery significantly predictor of *Bacteroides* and *Parabacteroides* abundance
	^42^	No differences in Shannon diversity index by mode of delivery		
	^45^		No association between mode of delivery and mode of delivery in RDA	

* Cluster 2 of taxonomic composition that was characterized by higher abundances of Gammaproteobacteria compared to Cluster 1.

† 2–4 weeks after introductions of enteral feeds.

‡ Supplementation with Dierol®.

GA: gestational age; MOM: mother’s own milk; OTU: operational taxonomic unit; PCA: principal component analysis; PPPROM: prolonged preterm premature rupture of membranes; PROM: premature rupture of membranes; PT: preterm; RDA: redundancy analysis.

##### Mode of delivery

A total of 21 studies reported associations between mode of delivery and the characteristics of the gut microbiota of PT infants, shown in (Table 2). One longitudinal study that followed PT infants from birth until discharge found that, over time, infants born via C-section had higher alpha diversity (Simpson diversity index) compared to vaginally delivered infants.^32^ However, a large number of studies reported no associations between mode of delivery and alpha diversity.^37–42^ Similarly, most of the studies found no differences in beta diversity by mode of delivery.^37,40,43–45^ Only two observational studies reported that mode of delivery explained 1.93%^37^ to 12%^41^ of the variation of beta diversity of the fecal microbiota of PT infants. It is important to note that results from Butcher et al. came from PT infants exclusively fed mother’s own milk (MOM).^37^ A cohort study analyzed the fecal microbiota composition of PT infants during early and late feeding stages, representing 1 to 2 weeks and 2 to 4 weeks after the introduction of enteral feeding, respectively. The authors found that mode of delivery was significantly associated with beta diversity (Unweighted UniFrac distances) only during the late feeding time points.^46^

Thirteen publications reported significant modifications in the taxonomic profile of PT infants depending on mode of delivery. A case-control study looking at the effect of histamine-2 receptor (H-2) blocker, found that Proteobacteria abundance was significantly lower in vaginally-delivered infants compared to infants born via C-section.^47^ Ho et al. reported that the abundance of Firmicutes was positively associated with birth via C-section.^35^ At class level, this same study reported a positive association between Gammaproteobacteria abundances and vaginal delivery at ≤ 2 weeks of postnatal age. This difference was mainly attributed to PT infants belonging to a cluster of colonization characterized by high abundances of Gammaproteobacteria.^35^ In accordance with this, La Rosa et al. found that the abundances of Gammaproteobacteria were negatively associated with C-section delivery only in infants born less than 26 weeks of GA.^48^ It was also reported by two different authors that the abundance of Bacilli was greater in PT infants delivered via C-section.^37,48^ Furthermore, vaginal delivery was positively associated with the abundances of *Bacteroides*,^41,44,49–51^ *Parabacteroides*,^44^ *Staphylococcus*,^38^ and *Enterobacteriaceae*,^52^ and was negatively associated with the abundances of *Enterobacter, Pantotea, Kluyvera, Erwinia, Klebsiella*^32^ and *Clostridium*.^52^ Differences between mode of delivery and gut microbiota composition seem to be more pronounced soon after birth, and diminish over postnatal time. A longitudinal study over the first 100 days of life of PT infants reported that during the first week after birth, vaginally delivered infants belonged to a bacterial cluster dominated by *Escherichia*, and infants born via C-section were more likely to associate with a cluster dominated by *Klebsiella*.^40^ Although these differences remained similar during the first four consecutive weeks of postnatal age, after the fifth week, both groups (vaginally delivered and C-section) showed similar patterns of colonization.^40^ Finally, a total of four studies found no differences in gut microbial composition and mode of delivery.^38,53–55^

#### Physiological factors

##### Ethnicity and sex

Few data exist regarding associations between ethnicity and sex and the gut microbial colonization of PT infants, as shown in (Table 3). A longitudinal observational study reported associations between race and the abundances of Firmicutes, and Gammaproteobacteria. The abundance of Firmicutes was positively associated with Latino ethnicity in PT infants with a colonization pattern low in Gammaproteobacteria. At ≤ 2 weeks postnatal age, Gammaproteobacteria abundance was positively associated with Latino ethnicity.^35^

**Table 3.** Physiological factors and gut microbiota composition of PT infants

Factor	Ref	Alpha diversity	Beta diversity	Taxonomy
***Ethnicity***	^35^			↑ Firmicutes*, ↑ Gammaproteobacteria in PT infants of Latino ethnicity
***Sex***	^33^	↑ Gini-Simpson diversity index in female PT infants	Sex explained 6% of the variance from Bray-Curtis dissimilarity index	
	^34^		No association between sex and gut microbiota composition (RDA)	
	^42^	No association between Shannon diversity index and sex		
***Weight and Growth***	^56^		Significant association between community composition and BW	
	^57^		Significant association between ELBW, VLBW and Bray Curtis distances and Unweight UniFrac distances	↑ Lactobacillales in ELBW infants fed PT formula at 28–30 weeks cGA† ↑ Clostridiales in VLBW infants fed PT formula over time
	^54^		Association between birth weight and microbiota composition (PLS-DA)	
	^58^	↓ Shannon diversity index in PT infants with growth failure‡		↑ *Staphylococcaceae, Bacteroideaceae* at 0–4 weeks postnatal age in PT infants with growth failure‡ ↑ *Enterobacteriaceae* and *Erysipelotrichaceae* at 3–9 weeks postnatal age in PT infants with postnatal growth failure‡ ↑ *Bacillaceae, Streptococcaceae, Peptostreptococcaceae, Veillonellaceae, Lachnospiraceae, Micrococcaceae, Tissierellaceae* and *Clostridiaceae* at 1–9 weeks postnatal age in PT infants with appropriate growth
***Gestational Age, Postnatal age and Corrected Gestational Age***	^65^			↑ *Comamonadaceae* at 2 days postnatal age ↑ *Enterobacteriaceae* at 10 days postnatal age ↑ *Bifidobacterium* at 30–90 days postnatal age
	^33^		GA explained ~2% of the variance from Bray-Curtis dissimilarity index	
	^68^			↑ *Bifidobacterium* positively correlated with postnatal age
	^56^		Significant association between community composition and GA, and cGA	Significant association between *Propionibacterium sp* and cGA
	^37^	↑ Shannon diversity index over time in PT infants fed MOM	GA explained 1.28% of the variation Postnatal age explained 7.73% of the variation in PT infants fed MOM	↑ Bacilli during early time points ↓ Bacilli after 21 days postnatal age in PT infants fed MOM ↑ Clostridia over time Gammaproteobacteria remained stable over time in PT infants fed MOM
	^32^			↓ *Staphylococcus, Escherichia* and *Shigella* over time ↑ *Veillonella, Streptococcus* and *Enterococcus* over time
	^50^	↑ Simpson diversity index over time ↓ Simpson diversity index in extremely PT infants compared to moderate and very PT infants§ ↑ Simpson diversity index in PT infants born ≥ 32 weeks GA across time No association between cGA and Simpson diversity index		↑ *Streptococcus* and *Bifidobacterium* in PT infants born >32 weeks GA ↑ *Bacteroides* and ↓ *Parabacteroides* in PT infants born >32 weeks GA at 6 weeks postnatal age ↑ *Pantoea* in moderate PT infants ↑ *Lactobacillus* and *Streptococcus* positively associated with cGA
	^62^		Significant association between postnatal age and UniFrac distances	↓ *Staphylococcus* over time
	^59^	↑ Shannon diversity index positively associated with GA at 10 days postnatal age		
	^17^			Delayed colonization with *Bifidobacterium*
	^60^	↑ Richness over time positively associated with postnatal age		
	^61^	↑ Shannon diversity index positively associated with postnatal age		↑ *Enterobacter aerogenes, Enterococcu*s spp., *Escherichia coli, Granulicatella* spp., *Klebsiella pneumoniae, Proteus, Serratia* and *Yersinia* at 21 days postnatal age
	^51^			↑ *Bacteroides* positively associated with postnatal age
	^57^	↑ Shannon diversity index positively associated with postnatal age and cGA (regardless diet)	Significant association between postnatal age and Bray Curtis distances	↑ Bacillales and Lactobacillales at 28–30 weeks cGA, particularly if formula-fed PT infants ↑ Enterobacteriales and Clostridiales in MOM and formula-fed PT infants
	^64^			↑ Bacilli at ≤29 weeks postmenstrual age ↑ Gammaproteobacteria at 28–36 weeks postmenstrual age ↑ Clostridia at 37 weeks postmenstrual age
	^35^	↑ Observed OTUs, phylodiversity, Shannon, Chao1 and Simpson diversity indices positively associated with postnatal age		↑ Gammaproteobacteria, Clostridia and Actinobacteria positively associated with postnatal age ↓ Bacilli over time
	^38^	↑ Observed OTUs over time		Progression from *Staphylococcus-Enterococcus* dominated gut microbiota during early points after birth to *Enterobacter* dominated, and finally *Bifidobacterium* dominated at later points
	^48^			↑ Bacilli in early time points (<28 days postnatal age) ↑ Clostridia in later time points (28 to >56 days postnatal age)
	^63^			↓ Proteobacteria over time in healthy PTI
	^66^			↑ *Staphylococcus* in meconium and stool at 1-week postnatal age ↑ *Enterococcus* at 2- and 3-weeks postnatal age ↑ Prevalence of *Serratia* in PT infants born <30 weeks GA ↓ *Propionibacterium, Lactobacillus plantarum, Streptococcus intermedius*, and *Streptococcus mitis* at 3 weeks postnatal age ↑ *Bacteroides splachnicus, Enterococcus, Clostridia, Veillonella, Clostridium difficile, E. coli, K. pneumoniae, Pseudomonas, Serratia* and *Yersinia* at 3 weeks postnatal age
	^67^			↑ *Enterococcus* dominated at <4 weeks postnatal age
	^43^		Significant association between postnatal age and Bray-Curtis dissimilarity index	↑ *Enterobacteriaceae* over time
	^52^			↑ *Bifidobacterium* and *Klebsiella* over time ↓ *Staphylococcus* and *Streptococcus* over time
	^40^	↑ Shannon diversity index over time		
	^55^			Transition over time from Gram-positive cocci dominated to *Enterobacteriaceae* and/or *Bifidobacteriaceae* Delayed colonization with *Bifidobacterium*
	^58^			↑ *Staphylococcaceae* in early time points at <5 weeks postnatal age in PT infants with postnatal growth failure‡ ↑ *Enterobacteriaceae* at 3–9 weeks postnatal age in PT infants with postnatal growth failure‡
	^42^		Significant association between day of life and Bray-Curtis dissimilarity index	*Enterobacter*: core microbiota in the first 60 days of postnatal age
	^45^			*Staphylococcus* and *Enterococcus* were part of the core microbiota of PT infants at 2 weeks postnatal age At 3 weeks postnatal age: ↑ *Enterococcus, Staphylococcus*, and *Enterobacter* in extremely PT infants ↑ *Bifidobacterium* in very PT infants

* When PTI belonged to Cluster 1, this was a cluster characterized by lower abundances of Gammaproteobacteria compared to Cluster 2.

† Gestational age at birth + postnatal age.

‡ Growth failure defined as weight below the 3^rd^ percentile according to the Fenton growth charts.

§ Extremely PT: born <28 weeks of gestation; Very PT: born 28–32 weeks of gestation; Moderate to late PT: born 32–37 weeks of gestation

BW: birth weight; cGA: corrected gestational age; ELBW: extremely low birth weight; GA: gestational age; MOM: mother’s own milk; OTU: operational taxonomic unit; PLS-DA: partial least squares discriminant analysis; PT: preterm; RDA: redundancy analysis; VLBW: very low birth weight.

In terms of differences in microbiota by infant sex, Cong et al. showed that alpha diversity, measured by the Gini-Simpson diversity index, was positively associated with female sex.^33^ As for beta diversity, sex explained 6% of the variance from the Bray-Curtis dissimilarity index.^33^ In contrast, two publications found no differences in alpha diversity,^42^ and beta diversity^34^ associated with infant’s sex.

##### Weight and growth

Four studies, summarized in (Table 3), reported differences in the gut microbial composition depending on weight and growth rate. Two observational studies found birth weight to be significantly associated with gut microbiota beta diversity of PT infants.^54,56^ Gregory et al. reported that, after birth, there were significant differences in beta diversity between PT infants with extremely low birth weight (ELBW, birth weight <1000 g) and PT infants with very low birth weight (VLBW, birth weight <1500 g).^57^ This same study, observed differences in the taxonomic composition by birth weight. However, these differences were primarily observed in infants fed PT formula. Across time, the abundances of Lactobacillales were higher in ELBW infants compared to VLBW infants. In contrast, the abundance of Clostridiales and Enterobacteriales was greater in VLBW across time compared to ELBW.^57^

A longitudinal study analyzed the association between growth and gut microbial colonization.^58^ The authors compared PT infants that presented growth failure (weight below the 3^rd^ percentile of the Fenton growth charts) at 40-weeks postmenstrual age and PT infants with appropriate growth. In the first nine weeks postnatal age, alpha diversity (Shannon diversity index) was lower in infants with growth failure.^58^ Infants that had growth failure had higher abundances of *Staphylococcaceae* and *Bacteroideceae* during the first weeks postnatal age, but during the third and ninth week of life, PT infants had greater abundance of *Enterobacteriaceae* and *Erysipelotrichaceae*.^58^ In the appropriate postnatal growth group, the authors found significant differences over time (1–9 weeks postnatal age) in bacteria of the family *Bacillaceae, Streptococcaceae, Peptostreptococcaceae, Veillonellaceae, Lachnospira-ceae, Micrococcaceae, Tissierellaceae* and *Clostridiaceae*.^58^ Furthermore, this same group created a gut microbiota maturity index to investigate its association with growth. The final model of this maturity index included the following discriminatory bacteria: Lactobacillales, *Peptostreptococcaceae, Clostridiaceaceae, Streptococcus, Staphylococcus, Veillonella, Enterococcus, Rahnella, Bifidobacterium*, and *Erwinia*.^58^ Even though the relative microbiota maturity index was positively correlated with postmenstrual age, infants with growth failure had significantly lower values of this index compared to infants with appropriate growth.^58^

##### Birth gestational age, postnatal age, and corrected gestational age

A total of 28 studies, shown in (Table 3), reported differences in diversity and composition of PT infants gut microbiota based on GA at birth, postnatal age, and corrected GA. Two longitudinal studies reported significant associations between GA at birth and different diversity indices.^50,59^ Dahl et al. analyzed the gut microbiota composition of PT infants at three different time points: 10 days, 4 months, and 1 year after birth. The authors found that Shannon diversity index was positively associated with GA at birth during the first 10 days postnatal age, even after controlling for exposure to antibiotics.^59^ Similar results were found by Chernikova et al. where after adjusting for postnatal age, antibiotic use, delivery mode and consumption of human milk, extremely PT infants (born <28 weeks GA) had significantly lower alpha diversity (measured by the Simpson diversity index) compared to very PT infants (born 28–32 weeks GA) and to moderate/late PT infants (born 32–37 weeks GA).^50^ Whereas alpha diversity was similar between very and moderate/late PT infants.^50^ Therefore, the authors created two groups of infants based on birth GA: infants born before 32 weeks GA, and those born ≥32 weeks GA. Infants born at a later age had higher Simpson diversity index compared to those born before 32 weeks of gestation.^50^ A large number of longitudinal observational studies reported that alpha diversity, measured by different indices, increases with postnatal age.^35,37,38,40,50,57,60,61^

Eight studies reported the effect of birth GA and postnatal age on beta diversity. Two longitudinal studies explored gut microbial colonization of PT infants based on type of feeding during the first days of postnatal age.^33,37^ Results showed that GA at birth explained 1.28%^37^ to 3%^33^ of the variance of the Bray-Curtis dissimilarity index. The former came from infants exclusively fed MOM,^37^ whereas the latter was independent of the feeding type.^33^ Four different observational studies looked at gut microbiota development of PT infants, with a follow-up period of the first 21,^62^ 30,^43^ or up to 60^42,57^ days of life. These studies found that postnatal age significantly associates with the community structure measured by UniFrac distances,^62^ and the Bray-Curtis dissimilarity index.^42,43,57^

Twenty-four studies included in this systematic review reported differences in taxonomic composition based on postnatal age. Mai et al. conducted a case-control study comparing PT infants with late onset sepsis to healthy PT infants.^63^ At phylum level, the authors found that in healthy infants, there is a decrease in the abundances of Proteobacteria over time.^63^ Evidence from multiple longitudinal studies suggests that during early time points after birth, there is an enrichment of Bacilli,^37,48,64^ which then decreases over time.^35^ This decrease in Bacilli coincides with an enrichment of Gammaproteobacteria,^35,64^ and Clostridia.^35,37,48,64^ In accordance, Gregory et al. showed that from 28–30 weeks of corrected GA, the gut microbiota is characterized by higher abundances of Bacillales and Lactobacillales.^57^ Following this, there is a significant decrease in Lactobacillales, particularly in infants fed PT formula.^57^ Around 31–33 weeks of corrected GA, in infants fed PT formula, there is a bloom of Enterobacteriales, and in infants fed PT formula plus MOM a bloom of Clostridiales.^57^ At family level, authors reported that during early-life time points (<5 weeks postnatal age) there are higher abundances of *Comamonadaceae*,^65^ and Gram-positive cocci^55^ such as *Staphylococcaceae*.^58^ Bacteria from the families of *Enterobacteriaceae*^43,55,58,65^ and *Bifidobacteriaceae*^55^ also increase their abundance over time.

At lower taxonomic rank, bacteria of the genera *Staphylococcus*,^38,45,66^ and *Enterococcus*^38,45,66,67^ are the main colonizers of PT infants gut during the first weeks of life (<4 weeks postnatal age). Zwittink et al. showed that at three weeks postnatal age, the mean relative abundance of *Staphylococcus* and *Enterococcus* was higher in extremely PT (<28 weeks GA) infants compared to very and moderate/late (32–37 weeks GA) PT infants.^45^ Following the first weeks of life, some studies report a decrease in the abundance of *Staphylococcus*,^32,52,62^ *Escherichia-Shigella*,^32^ *Streptococcus*,^52^ and *Parabacteroides*.^50^ Furthermore, there is a positive association between postnatal age and the presence and/or abundance of specific bacteria, including *Anaerobiospirillum*,^66^ *Haemophilus*,^66^ *Veillonella*,^32,66^ *Lactobacillus*,^50^ *Bacteroides*,^51^ Clostridia,^66^ *Serratia*,^61,66^ *Yersinia*,^61,66^ *Pseudomonas*,^66^ *Klebsiella*,^52^ *Granulicatella*,^61,66^ *Proteus*,^66^ *Propionibacterium*,^56^ and *Enterobacter*.^38^ Zhou et al. reported that in their study population, *Enterobacter* was a member of the core microbiota of PT infants over the first 60 days of postnatal age.^42^ However, these results are still not consistent. Moles et al. found a decrease in the abundance of *Propionibacterium* from meconium samples to stool samples during the third week of life.^66^ It is important to highlight that although studies report an increase in the abundance of *Bifidobacterium* over time,^38,52,65,68^ evidence converges in that the colonization with this obligate anaerobe is delayed in PT infants.^17,55^

The colonization pattern across time in PT infants is also affected by GA at birth. Chernikova et al. reported that infants born >32 weeks GA are colonized with greater abundances of *Streptococcus* and *Bifidobacterium* than those born ≤32 weeks GA.^50^ At 6-weeks of postnatal age, infants born >32 weeks GA had a higher number of members from the genera *Bacteroides* and lower abundance of *Parabacteroides*.^50^ This same study also reported that the abundance of *Pantoea*, a bacteria of the family *Enterobacteriaceae*, was higher in moderate/late PT infants (32–37 weeks GA), even after adjusting for other exposures including postnatal age.^50^ Another longitudinal study found that at three weeks postnatal age, extremely PT infants (<28 weeks GA) had higher abundances of *Enterobacter*, whereas very PT infants (28–32 weeks GA) harbored higher abundances of *Bifidobacterium*.^45^

Less data exists regarding the relationship between specific species and postnatal age. Two longitudinal studies analyzed the gut microbiota of PT infants at species level utilizing human intestinal tract chip analysis^61^ or PCR-amplified 16S rRNA fragments.^66^ There was a positive association between postnatal age and the abundance of *Enterobacter aerogenes*,^61^ *Bacteroides splachnicus*,^66^ *Escherichia col*i,^61^ *Clostridium difficille*,^66^ and *Klebsiella pneumoniae*.^61,66^ Whereas, over time, there was a decrease in *Prevotella tannerae, Lactobacillus plantarum, Streptococcus intermedius*, and *Streptococcus mitis*.^66^

#### Pharmacological factors

##### Antibiotics

Twenty-two studies reported the effect of antibiotics on the gut bacterial communities of PT infants, presented in (Table 4). As expected, antimicrobial agents reduced the gut bacterial diversity.^32,34,42,56,60,69,70^ Two observational studies determined that the duration of antibiotic exposure was significantly associated with the reduction in microbial diversity.^34,71^ The decrease in alpha diversity was similar in PT infants that were exposed to short antibiotic treatment, ranging from ≤ 3 days^34^ to ≤ 7 days,^71^ or exposed to longer treatment (≥ 5 days or > 7 days).^34,71^ Furthermore, the reduction in alpha diversity seems to be only temporal. Several studies reported that a decrease in diversity indices like observed OTUs, Simpson, Shannon, Chao1, and phylogenetic diversity remains significant only within the first week after the use of antibiotics.^34,42,56,70^ In fact, diversity tends to recover after the cessation of antibiotic treatment.^32,34^ Nonetheless, some studies reported no effect of antibiotics in diversity metrics^38^ or opposite results^41^ than those previously described in this review. Wandro et al. conducted a longitudinal study of VLBW (<1500 g) infants and found decrease Shannon diversity index in PT infants with no record of antibiotic use.^41^

**Table 4.** Pharmacological factors and gut microbiota composition of PT infants

Factor	Ref	Alpha diversity	Beta diversity	Taxonomy
***Antibiotics***	^65^			↑ *Bifidobacteriaceae, Streptococcaceae Comamonadaceae, Staphylococcaceae* and unclassified Bacilli at 30 days postnatal age in PT infants never exposed to antibiotics
	^56^	↓ Diversity during or within 5 days of antibiotic use		
	^37^			↑ Gammaproteobacteria and ↓ Clostridia with higher exposure to antibiotics in PT infants fed MOM
	^32^	↓ Simpson diversity index with use of antibiotics		
	^50^			↓ *Bifidobacterium* and *Bacteroides* with antibiotic use after birth
	^33^		Antibiotic use in the first 48–72 h explained 2–3% of the Bray-Curtis dissimilarity index	
	^53^			No differences between short and prolonged* exposure to antibiotic during week 1 postnatal age ↓ *Lactobacillus* and *Veillonella* at 4 months postnatal age when exposed to broad-spectrum antibiotic therapy
	^60^	↓ Species richness with the use of antibiotics		↑ *Staphylococcus epidermis* after meroprenem use ↑ *Klebsiella pneumoniae* after ticarcillin-clavulanate use ↑ *Escherichia coli* with cefotaxime use
	^51^			No association of antibiotic exposure with *Bacteroides* abundance
	^64^			Association between Gammaproteobacteria abundance and antibiotic use
	^38^	No significantly difference in bacteria richness after antibiotic use		↓ *Enterococcus* with aminoglycoside use ↑ *Enterococcus* with vancomycin use ↓ *Bifidobacterium* with aminoglycoside or vancomycin use Changes were temporal, microbiota recovered within days after treatment termination
	^48^			↓ Clostridia in PT infants born <28 weeks GA with antibiotic use ↑ Gammaproteobacteria in PT infants born >26 weeks GA with antibiotic use
	^73^			16–17% less chance of *Veillonellaceae* colonization with every day increase in use of antibiotics
	^74^			*Enterococcus faecalis, Enterococcus faecium, Staphylococcus aureus, E. col*i and *K. pneumoniae* showed resistance to antibiotics At 2-years postnatal age, fecal samples were susceptible to antibiotics
	^43^		Significantly association between use of antibiotics and UniFrac distances at week 1 postnatal age	
	^41^	↓ Shannon diversity index in PT infants with no antibiotic use		
	^69^	↓ Bacteria count in PT infants on day 30 after receiving antibiotics		
	^42^	↓ Observed OTUs and Shannon diversity index within 5 days after receiving antibiotics		
	^70^	↓ Shannon diversity index on day 7 after antibiotic use		↑ Bacteroides and Actinobacteria on day 3 after treatment with penicillin-moxalactam and piperacillin-tazobactam ↑ *Sphingomonas, Bacteroides, Lactobacillus* on day 3 after treatment with penicillin-moxalactam use and piperacillin-tazobactam ↓ *Clostridium* on day 3 after treatment with penicillin-moxalactam use ↑ *Enterococcus* and ↓ *Klebsiella* on day 7 after treatment with piperacillin-tazobactam use ↑ *Escherichia-Shigella* with penicillin-moxalactam use
	^71^	No differences in Shannon diversity index between PT infants with low or high exposure to antibiotics†		↑ Betaproteobacteria in infants with high† exposure to antibiotics ↓ *Bifidobacterium* in infants with high† exposure to antibiotics
	^34^	No difference between short and long antibiotic treatment‡ ↓ Chao 1 and PD after antibiotic use during week 1 postnatal age Recovery of diversity after secession of treatment	Duration of antibiotic use explained 3.6% of the variation of fecal microbiota composition (RDA)	↓ *Enterobacteriaceae* with antibiotic use ↓ *Bifidobacterium* after short antibiotic use during the first 3-weeks postnatal age‡ ↓ *Bifidobacterium* after long antibiotic use during the first 6-weeks postnatal age‡ ↑ *Enterococcus* with antibiotic use
	^45^		Duration and number of antibiotics administrated explained 25.6% of the variation of fecal microbiota composition (RDA)	
***Other medications***	^64^			↑ *Bifidobacterium* with H2-blockers at >33 weeks postmenstrual age
	^47^	↓ Shannon diversity index in PT infants that received H2-blockers		↑ Proteobacteria and ↓ Firmicutes in PT infants that received H2-blockers ↑ Gammaproteobacteria and *Enterobacteriaceae* in PT infants that received H2-blockers

* Short: ≤ 72 h; prolonged: >72 h.

† Low exposure: ≤ 7 days; High exposure: > 7 days.

‡ Short antibiotic treatment: ≤ 3 days; long antibiotic treatment: ≥ 5 days.

GA: gestational age; H2: histamine-2 receptor; OTU: operational taxonomic unit; PD: phylogenetic diversity; PT: preterm; RDA: redundancy analysis.

Several studies found significant associations between the use of antibiotics and beta diversity of gut microbiota from PT infants.^33,34,43,45^ Cong et al. observed that antibiotic use within the first 48–72 hours after birth explained ~3% of the variation from the Bray-Curtis dissimilarity index.^33^ Evidence from two studies conducted by Zwittink et al. described a strong association between antibiotic treatment and beta diversity.^34,45^ The duration of the use of antibiotics, whether it was less than three days or more than five days, explained 3.6% of the variation of the gut microbiota composition.^34^ Furthermore, up to 25.6% of the variance of these bacterial communities was explained when more antibiotic-related factors were taken into consideration, such as duration and number of antibiotics that were administrated.^45^

Changes in diversity induced by antimicrobial agents in PT infants subsequently influence taxonomic composition of the fecal microbiota, with some bacteria decreasing while others blooming. Specifically, there was a positive association between the exposure to antibiotics and the abundance of Gammaproteobacteria^37,48,64^ and Betaproteobacteria,^71^ while there was a negative association with bacteria from the class Clostridia.^48,72^ A study found that, at 30 days postnatal age, PT infants who were never exposed to antibiotics, had higher abundances of *Bifidobacteriaceae, Streptococcaceae, Comamonadaceae, Staphylococcaceae*, and unclassified Bacilli compared to infants that have been previously exposed to antibiotics.^65^ Millar et al. reported that by each successive day of antibiotic usage in PT infants, there is 16% to 17% less chance of colonization with *Veillonallaceae*.^73^ In accordance with this, another study reported that exposure to antibiotics reduces the presence of bacteria from the genus *Veillonella*.^53^ Different studies reported a negative association between the exposure to antibiotics and the abundance of *Enterobacteriaceae*,^34^ *Lactobacillus*,^53^ *Bifidobacterium*,^34,50,71^ and *Bacteroides*;^50^ and a positive association between antibiotic use and the abundance of *Enterococcus*.^34^ However, a study looking specifically at *Bacteroides* gut colonization in PT infants found no association between antibiotic use and the abundance of this bacteria.^51^

Importantly, microbiota modifications caused by antibiotics might depend on the type of antibiotic used. Gibson et al. assessed the effect of different antibiotics, including meropenem, cefotaxime, ticarcillin/clavulanate, ampicillin, vancomycin, and gentamicin. They reported an increase in *Staphylococcus epidermis* after the use of meropenem (beta-lactamase inhibitor), *Klebsiella pneumoniae* after the use of Ticarcillin-Clavulanate (combined extended-spectrum penicillin with a beta-lactamase inhibitor), and *Escherichia coli* with the use of cefotaxime, a broad spectrum cephalosporin antibiotic.^60^ All of these medications exert their antibiotic effects by affecting cell wall synthesis, or by causing cell death. A similar study conducted by Zhu et al. reported an increase in bacteria of the phylum Bacteroides and Actinobacteria with the use of penicillin-moxalactam (an oxacephem antibiotic usually grouped with the cephalosporins) and with the use of piperacillin-tazobactam (penicillin with a beta-lactamase inhibitor).^70^ With penicillin-moxalactam, PT infants had greater abundances of *Sphingomonas, Bacteroides*, and *Lactobacillus*, and a decrease in *Clostridium*.^70^ Korpela and collaborators found a decrease in *Bifidobacterium* abundances when an antibiotic of the class aminoglycosides or vancomycin were used.^38^ This same study also described that when antibiotics of the class of aminoglycosides were administered, the abundance of *Enterococcus* decreased. In contrast, when vancomycin was used, the presence of *Enterococcus* was higher.^38^ Although the taxonomic modifications were significant, these were only temporal, and the microbiota structure recovered within days after the cessation of antibiotic treatment.^38^

Even though antibiotics are used to treat or reduce the presence of pathogenic bacteria in PT infants, their efficacy could be blunted by the presence of antibiotic-resistant bacteria. Moles et al. evaluated the gut colonization of PT infants by antibiotic-resistant bacteria during the first week of life and at 2-years of age.^74^ Bacteria isolates obtained from stool samples were assessed for antibiotic susceptibility using agar dilution assays. This assay consists in platting the isolates in agar medium with antibiotics and measuring the diameters of the colonies that were exposed to the antibiotic.^75^ The authors also performed bacteria identification at species level using MALDI-TOF spectrometry. In the early postpartum period, PT infants were colonized by a number of antibiotic-resistant bacteria including *Enterococcus faecalis, Enterococcus faecium, Staphylococcus aureus, Escherichia coli*, and *Klebsiella pneumonia*.^74^ However, by 2-years of age, these same bacteria showed antibiotic susceptibility.^74^

##### Other medications

Only a few studies have evaluated the effect of medications other than antibiotics on the gut colonization of PT infants, as shown in (Table 4). Gupta et al. conducted a case-control/cross-sectional study of infants who received H2-blockers vs. infants who did not received this medication.^47^ There was a decrease in the alpha diversity (measured by Shannon diversity index) in PT infants that were exposed to H2-blockers compared to those not exposed to this medication.^47^ Taxonomically, after the administration of H2-blockers, there was a significant decrease in Firmicutes accompanied by an increase of Proteobacteria. At lower taxonomic ranks, infants that were exposed to H2-blockers had increased abundance of Gammaproteobacteria and *Enterobacteriaceae*.^47^ Additionally, an observational study found a positive association between the use of H2-blockers and the abundance of *Bifidobacterium* at a later time point of the follow-up period (>33 weeks postmenstrual age).^64^

#### Dietary factors

##### Macronutrients

Several studies, summarized in (Table 5), have reported modifications of the gut microbiota of PT infants based on macronutrient composition, type of milk consumed, and use of fortifiers. A longitudinal study following PT infants during the hospitalization period in the NICU found that the ratio of grams of enteral lipids to total calories (g/kcal) was positively associated with the abundance of Actinobacteria, the ratio of enteral protein (g/kcal) with Firmicutes abundance, and ratio enteral carbohydrate (g/kcal) with abundance of Actinobacteria, Proteobacteria, and Firmicutes.^64^ At 33-weeks postmenstrual age, there was an increase of *Bifidobacterium* abundance associated with greater ratio of enteral lipid intake whereas, and higher ratio of enteral protein intake was associated with reduced *Bifidobacterium*.^64^

**Table 5.** Dietary factors and gut microbiota composition of PT infants

Factor	Ref	Alpha diversity	Beta diversity	Taxonomy
***Macronutrients***	^64^			↑ Actinobacteria, and Proteobacteria with higher lipid intake* ↑ Firmicutes with higher protein intake† ↑ Actinobacteria, Proteobacteria and Firmicutes with higher carbohydrate intake‡ ↑ *Bifidobacterium* with lipid intake at >33 weeks postmenstrual age ↓ *Bifidobacterium* with protein intake at >33 weeks postmenstrual age
	^76^	↑ Shannon diversity and Inverse Simpson indices over time in PT infants with HF-PUFA enteral supplementation		↓ Proteobacteria and ↑ Actinobacteria in PT infants with HF-PUFA enteral supplementation ↑ *Corynebacterium, Geobacillus, Erwinia* in PT infants with HF-PUFA enteral supplementation ↑ *Escherichia-Shigella, Salmonella, Serratia, Pantoea, Clostridium, Tatumella, Streptococcus, Cedeceae* and *Citrobacter* in control group
***Milk and Fortifiers***	^68^			↑ *Staphylococcus* in PT infants consuming MOM with high content of *Staphylococcus* ↑ *Bifidobacterium* in PT infants consuming MOM with high content of *Rothia, Enterococcus* and *Streptococcus*
	^37^			Bacilli, Clostridia and Gammaproteobacteria compromised >90% of bacteria abundance over time in PT infants fed MOM Low levels of *Bifidobacterium* in PT infants fed MOM No changes in gut microbiota composition after fortification of MOM with BMF
	^46^	↑ Chao1 diversity index in PT infants fed MOM + BMF compared to MOM + PT formula or PT formula alone		↑ Proteobacteria in PT infants fed MOM + BMF ↑ *Terrisporobacter* and *Peptoclostridium* in formula-fed infants ↓ *Veillonella* in PT infants fed MOM + BMF
	^50^	No association between consumption of MOM and/or DHM with alpha diversity		↓ *Lactobacillus* in PT infants fed MOM and/or DHM
	^33^	↑ Gini-Simpson diversity index in PT infants fed MOM compared to DHM, PF or the combination of two different types of milk. No association between human milk (MOM and/or DHM) fortification with BMF and alpha diversity	Feeding type explained 11% of the variance of Bray-Curtis dissimilarity index	↑ Clostridiales, Lactobacillales and Bacillales in PT infants fed MOM ↑ *Enterobacteriales* in PT infants fed DHM, PT formula, and DHM + PT formula ↑ Bifidobacteriales in PT infants fed MOM and MOM + PT formula
	^59^	↑ Shannon diversity index in early PT formula introduction (<10 days of age)		↓ Firmicutes and ↑ Proteobacteria at 10 days postnatal age in exclusively breastfed PT infants compared to full-term infants
	^60^	↑ Species richness in PT infants fed human milk (MOM and/or DHM)		
	^57^		Association between different types of milk and Bray-Curtis distances	↑ Lactobacillales, Enterobacteriales and Clostridiales in formula-fed PT infants ↑ Clostridiales in VLBW PT infants fed MOM *Citrobacter, Clostridium, Ruminococcus* and *Negativicoccus*, best discriminators of PT infants fed MOM *Streptococcus, Bacillus* and *Anaerococcus*, best discriminators of PT infants fed PT formula
	^48^			↑ Gammaproteobacteria at 28 days postnatal age and at 28 to >56 days postnatal age with higher MOM consumption
	^77^	No association between type of milk consumed and Simpson diversity index at >7 days postnatal age		
	^78^		Association between type of milk consumed and Bray-Curtis dissimilarity index and UniFrac distances	↑ *Bifidobacterium, Acitenobacter* and *Haemophilus* in PT infants fed MOM ↑ *Staphylococcus, Clostridium, Coprococcus, Aggregatibacter* and *Lactobacillus*, in PT infants fed DHM ↑ *Blautia, Streptococcus, Acidaminococcus, Rothia* and *Dorea* in formula-fed PT infants
	^79^		Association between type of milk consumed and gut microbiota composition	
	^80^			↓ *Staphylococcus aureus, Staphylococcus hominis, Staphylococcus lugdunensis* in PT infants fed MOM compared to full-term infants
	^28^			↓ *Bifidobacterium* in PT infants fed MOM + BMF ↑ Gammaproteobacteria and ↓ Bacillales in PT infants fed MOM + HMF
	^81^			↑ *Lactobacillaceae* and ↓ Gammaproteobacteria when consuming MOM of secretor mothers
	^30^	↓ Shannon diversity index in PT infants fed PT formula compared to MOM		
	^41^	No association between type of milk consumed and Shannon diversity index		
	^45^		No association between human milk consumption and gut microbiota composition	
	^34^		No association between human milk consumption and gut microbiota composition	
***Prebiotics and/or probiotics***	^27^	↑ Shannon diversity index after Infloran® supplementation		↑ *Lactobacillus* and *Bifidobacterium* after supplementation with Infloran®, effect remained after treatment
	^82^			↓ *Enterobacteriaceae* in groups receiving 5, 10, and 15 g/day of honey ↑ *Bifidobacterium* after 2 weeks of supplementation with honey (regardless of dose) ↑ *Lactobacillus* only in group receiving 10 g/day of honey
	^83^			↑ *Lactobacillus* with supplementation of GOS + FOS but no difference compared to control group
	^53^			↑ *Bifidobacterium* and *Lactobacillus* in PT infants supplemented with Infloran® at 7 days postnatal age ↑ *Escherichia*, ↓ *Veillonella* and *Streptococcus* in PT infants supplemented with Infloran® at 28 days postnatal age
	^84^			↑ *Bifidobacterium* in PT infants supplemented with *Bifidobacterium breve* and combination of *B. breve* + *Bifidobacterium infantis* + *Bifidobacterium longum* ↓ *Enterobacteriaceae* in PT infants supplemented *B. breve* + *B. infantis* +*B. longum*
	^73^	No differences in Simpson diversity index between PT infants supplemented with *B. breve* strain BBG-001		
	^85^			↓ *Clostridium histolyticum* in PT infants receiving *Lactobacillus rhamnosus* supplementation
	^86^			↑ *Bifidobacterium* and *Lactobacillus* in PT infants supplemented with *B. longum* BB536 + *L. rhamnosus* GG
	^28^			↑ Clostridia with increasing doses of GOS or HMOs supplementation
	^29^			↑ *Enterobacteriaceae* and *Clostridiceae* in non-responders (low *Bifidobacterium* colonization) to the supplementation with *B. breve*
	^30^	↑ Shannon diversity index in PT infants fed PT formula with *Bifidobacterium animals* subsp. *lactis* supplementation		↓ *Bifidobacterium* in PT infants fed PT formula with *Bifidobacterium lactis* supplementation (no dose response) ↑ *Bifidobacterium* in PT infants fed PF with *B. infantis*, peaking after dose 4 (dose response) ↑↑ *Bifidobacterium* in PT infants consuming MOM supplemented with *B. infantis* ↓ *Proteobacteria* with supplementation of *B. infantis*
	^87^			↑ *Bifidobacterium* and *Lactobacillus* over time with ProBioPlus DDS® supplementation ↓ Gram-negative bacteria with Culturelle® supplementation
	^69^			No changes in colonization after supplementation of GOS + FOS + AOS
	^44^	↓ Simpson diversity index after Dierol® supplementation No differences when comparing with placebo	No association between supplementation with Dierol® (before and after) and gut microbiota composition	↓ *Enterococcus, Pseudomonas* and ↑ *Veillonella, Clostridium* and *Bifidobacterium* with Dierol® supplementation

* Ratio of grams of lipids to total enteral calories (g/kcal)

† Ratio of grams of protein to total enteral calories (g/kcal)

‡ Ratio of grams of carbohydrates to total enteral calories (g/kcal)

¶ Mothers that express 2′-fucosyltransferase and produce milk containing 2′-fucosyllactose and lactodifucotetraose

Infloran®: *Lactobacillus acidophilus* + *Bifidobacterium. bifidum*

ProBioPlus DDS: *Lactobacillus acidophilus* + *Bifidobacterium longum* + *Bifidobacterium bifidum* + *Bifidobacterium infantis* + inulin

Culturelle: *Lactobacillus rhamnosus* GG + inulin

Dierol®: *Saccharomyces. Boulardii*

AOS: acidic oligosaccharides; DHM: donor human milk; FOS: fructooligosaccharides; GOS: galactooligosaccharides; HF-PUFA: high-fat polyunsaturated fatty acids; HM: human milk; HMF: human milk fortifier; HMOs: human milk oligosaccharides; MOM: mother’s own milk; PF: preterm formula; PMA: postmenstrual age; PT: preterm.

Younge et al. conducted a randomized controlled trial (RCT) to test the effect of enteral supplementation of high-fat polyunsaturated fatty acid (HF-PUFA) from fish oil and safflower oil on the gut microbiota of PT infants.^76^ There were no differences in the first week after supplementation with HF-PUFA, but over time, alpha diversity (measured by Shannon and inverse Simpson indices) was higher in infants that received HF-PUFA supplementation.^76^ At phylum level, those receiving the HF-PUFA intervention had a lower abundance of Proteobacteria and higher abundance of Actinobacteria than those without the intervention.^76^ There were further differences at the genus level, and these differences were categorized as early (1–9 weeks), mid (2–9 weeks), or late (4–9 weeks) changes after treatment initiation. Some of the early changes were a decrease in *Escherichia-Shigella* and *Salmonella* in the HF-PUFA, and an increase in the abundance of *Corynebacterium* and *Geobacillus*. At 2–9 weeks after initiation of HF-PUFA, supplementation, there was a significant increase in the relative abundance of *Erwinia* and decreases in *Serratia, Pantoea, Clostridium, Tatumella*, and *Streptococcus*. Lastly, a reduction in fecal *Cedecea* and *Citrobacter* in the HF-PUFA group was reported as a late change.^76^

##### Milk and fortifiers

Twenty studies reported associations related to milk and/or fortifier administration to PT infants and the structure of their gut microbiota, shown in (Table 5). One study reported an increase in the alpha diversity with the consumption of exclusively MOM, compared to donor human milk (DHM), PT formula or the combination of two different types of milk (MOM + DHM, MOM + PT formula, or DHM + PT formula).^33^ Gibson et al described an increase in species richness with the consumption of human milk (MOM, DHM or the combination of both).^60^ Another study reported that the combination of MOM with a bovine milk-based fortifier, two to four weeks after the introduction of enteral feeds, significantly increased alpha diversity compared to PT infants fed PT formula alone or in combination with MOM.^46^ Underwood et al. found a lower Shannon diversity index in infants fed PT formula compared to those fed MOM.^30^ Additionally, one study reported that introduction of PT formula before 10 days postnatal age was positively associated with Shannon diversity index.^59^ Nonetheless, some studies found no differences in alpha diversity based on the type of diet, whether it was human milk (MOM and/or DHM),^41,50,77^ PT formula,^77^ the combination of human milk and PT formula,^41^ or the supplementation of human milk with a bovine milk-based fortifier.^33^

Six different studies described the effect of milk and fortifiers on beta diversity. There was a positive association between feeding type (MOM, DHM, and PT formula) and beta diversity, measured by Bray-Curtis dissimilarity index^57,78^ and UniFrac distances.^78^ Another study comparing only human milk vs. PT formula found an association between these feeding exposures and the gut microbiota structure.^79^ Cong et al. compared the effect of different types of milks and found that up to 11% of the variance in the Bray-Curtis dissimilarity index could be explained by the feeding type.^33^ However, some studies found no association between human milk consumption and the gut microbiota beta diversity of infants born preterm.^34,45^

Taxonomically, studies found a positive association between the abundance of Proteobacteria with MOM consumption^59^ and the fortification with a bovine milk-based fortifier,^46^ as well as a negative association between MOM consumption and the abundance of Firmicutes.^59^ A study conducted by La Rosa et al. reported that across time, there is a linear relationship between the abundance of Gammaproteobacteria and MOM consumption.^48^ et al. found a decrease in the abundance of *Lactobacillus* with exposure to human milk (MOM and/or DHM).^50^ Butcher et al. followed PT infants that were exclusively fed MOM and identified that these infants were mainly colonized by Bacilli, Clostridia, and Gammaproteobacteria, with very low levels of *Bifidobacterium*.^37^ In contrast, different studies showed that exposure to MOM was associated with greater abundances of bacteria of the class Lactobacillales,^33^ Bacillales,^33^ Bifidobacteriales,^33^ and Clostridiales,^33,57^ and higher abundance of the genera *Bifidobacterium, Acinetobacter* and *Haemophilus*.^78^ One study compared the gut colonization specifically by species of the genus *Staphylococcus* in PT infants exclusively fed MOM.^80^ Results showed that infants fed MOM had a lower presence of *Staphylococcus aureus, Staphylococcus hominis*, and *Staphylococcus lugdunesis* compared to full-term infants.^80^ Some of these differences could be attributed to the composition of human milk. Underwood et al. reported that PT infants consuming MOM of secretor mothers (expressing 2′-fucosyltransferase) that produce milk containing the human milk oligosaccharides (HMOs), 2′-fucosyllactose and lactodifucotetraose, had a lower abundance of Gammaproteobacteria and higher abundance of *Lactobacillaceae*.^81^ Another observational study analyzed the gut microbial composition of PT infants and of the milk they were fed.^68^ The authors reported that when infants consumed MOM with a high abundance of *Staphylococcus*, they harbored a gut microbiota rich in *Staphylococcus*.^68^ In contrast, infants that consumed MOM high in *Rothia, Enterococcus*, and *Streptococcus* developed a gut microbiota with higher abundances of *Bifidobacterium*.^68^

Studies also reported differences in the gut microbiota based on DHM and PT formula consumption. Preterm infants that were fed DHM had higher abundances of Enterobacteriales,^33^ *Staphylococcus, Clostridium, Coprococcus, Aggregatibacter*, and *Lactobacillus*.^78^ If infants were exclusively fed PT formula, they had a greater abundance of Lactobacillales,^57^ Enterobacteriales,^33,57^ and Clostridiales^57^ compared to those fed human milk. At genus level, PT formula consumption was positively associated with the abundance of *Blautia, Streptococcus, Acidaminococcus, Rothia, Dorea*,^78^ *Terrisporobacter* and *Peptoclostridium*.^46^ An observational study concluded that the best discriminators of the gut microbiota of PT infants fed MOM were bacteria of the genus *Citrobacter, Clostridium, Ruminococcus*, and *Negativicoccus*, whereas the best discriminators of infants consuming PT formula were *Streptococcus, Bacillus*, and *Anaerococcus*.^57^

Although there are differences in gut microbiota composition depending on the type of milk consumed, PT infants are likely to be fed more than one type of milk at a time. As previously mentioned, a study evaluating different feeding patterns found that when infants were fed DHM and/or PT formula, they had increased levels of Enterobacteriales compared to other feeding groups and combinations.^33^ Infants consuming MOM in combination with PT formula, had the highest enrichment of Bifidobacteriales.^33^ Furthermore, studies also observed differences in the gut microbiota depending on the type of fortification to human milk. Cai et al. described a decrease in *Veillonella* with a bovine milk fortifier in infants consuming MOM.^46^ Another study found that PT infants fed MOM with human milk-based fortifier had a higher abundance of Gammaproteobacteria and a lower abundance of Bacillales.^28^ Additionally, an increase in *Bifidobacterium* was observed if PT infants were fed MOM with the addition of a human milk-based fortifier.^28^ In contrast, Butcher et al. did not find changes in the gut microbiota of PT infants when they received MOM fortified with a bovine milk-based fortifier.^37^

##### Prebiotics and probiotics

Fourteen studies described the effect of prebiotics or probiotics in the gut microbiota of PT infants, presented in (Table 5). These studies were focused on supplementation with prebiotics,^28,69,82,83^ probiotics,^27,29,30,44,84–86^ or both.^87^ The prebiotics tested on PT infants were fructooligosaccharides (FOS) from clover honey,^82^ galactooligosaccharides (GOS) + FOS,^83^ GOS vs. HMOs,^28^ or GOS + FOS + acidic oligosaccharides (AOS).^69^ Publications related to prebiotic supplementation did not report differences in alpha or beta diversity subsequent to supplementation. However, several taxonomic differences were found with prebiotic supplementation. When PT infants received FOS alone, there was a decrease in *Enterobacteriaceae* and an increase in *Bifidobacterium* and *Lactobacillus*.^88^ With the supplementation of GOS + FOS, the abundance of *Lactobacillus* increased over time; however, the abundance of *Lactobacillus* was not significantly different than the control group.^83^ Despite the changes mentioned above, Westerbeek et al. did not find any significant changes in the gut colonization of PT infants after intervention with a mixture of GOS + FOS + AOS.^69^ Underwood et al. reported an increase in Clostridia with increasing doses of either GOS or HMOs added to PT formula compared to PT formula without these prebiotics.^28^

The probiotics that were supplemented in the studies included in this review include: Infloran® (*Lactobacillus acidophilus* + *Bifidobacterium bifidum*),^27,53^ a mixture of *Bifidobacterium breve* + *Bifidobacterium longum* subsp. *infantis* + *Bifidobacterium longum* subsp. *Longum*,^84^ single strain administration of *Bifidobacterium breve*,^73,84^ *Bifidobacterium longum* BB536 + *Lactobacillus rhamnosus* GG,^86^ *Lactobacillus rhamnosus*,^85^ *Bifidobacterium breve* M16-B,^29^ *Bifidobacterium longum* subsp. *infantis* or *Bifidobacterium animals* subsp. *lactis*,^30^ or Dierol® (*Saccharomyces boulardii*).^44^ After supplementation with Infloran®, there was an increase in the alpha diversity, measured by Shannon diversity index.^27^ Both publications supplementing Infloran® to PT infants reported an increase in *Lactobacillus* and *Bifidobacterium* after treatment.^27,53^ Additionally, in PT infants supplemented with Infloran®, there was an increase *Escherichia*, along with a decrease in *Veillonella*, and *Streptococcus* at 28 days postnatal age.^53^ The treatment with *B. breve* showed no impact on the alpha diversity (measured by Simpson diversity index) after supplementation.^73^ Administration of *B. breve* alone or in with *B. infants* + *B. longum* increased the abundances of *Bifidobacterium* in the gut of PT infants.^84^ When infants received *B. longum* + *L. rhamnosus* there was an increase in *Bifidobacterium* as well as in *Lactobacillus*.^86^ Underwood et al. found an increase in the Shannon diversity index when formula-fed PT infants received *B. animalis* subsp. *lactis*.^30^ This study also found an increase in *Bifidobacterium* after supplementation with *B. longum* subsp *infantis* in infants consuming PT formula; however, this increase was even greater in PT infants receiving the supplementation while consuming MOM.^30^ There was no dose-response in *Bifidobacterium* abundance in the group supplemented with *B. animalis* subs *lactis*.^30^ Overall, the supplementation with probiotics decreased the presence of Proteobacteria,^30^ *Enterobacteriaceae*,^84^ and *Clostridium histolyticum*.^85^ A study found that after supplementation with *B. breve*, the gut microbiota of some PT infants had less than 6% abundance of *Bifidobacterium*; these infants were described as non-responders.^29^ Additionally, these infants actually had increased abundances of *Enterobacteriaceae* and *Clostridiaceae*.^29^ After supplementation with Dierol®, there was a decrease in the alpha diversity (measured by Simpson diversity index) overtime. However, this reduction was not significantly different than the one showed in the placebo group.^44^ The authors found no association between probiotic supplementation with Dierol® and the gut microbiota beta diversity. Dierol® supplementation was associated with an increase in *Veillonella, Clostridium*, and *Bifidobacterium*, as well as a decrease in *Enterococcus* and *Pseudomonas*.^44^

One study aimed to analyze the effect of the combination of prebiotic and probiotics. Preterm infants were exposed to Culturelle® (*L. rhamnosus* GG + inulin) or to ProBioPlus DDS® (*L. acidophilus* + *B. longum* + *B. bifidum* + *B. infantis* + inulin). Over four weeks of supplementation, infants exposed to Culturelle® showed a decrease in Gram-negative bacteria, whereas supplementation with ProBioPlus DDS® significantly increased *Bifidobacterium* and *Lactobacillus* over the same 4-week period.^87^

#### Environmental factors

##### NICU environment

Recently, studies have aimed to analyze the effect of the hospital and NICU environment on the gut colonization of PT infants. Four studies, summarized in (Table 6), reported associations related to the gut microbiota and the hospitalization period. Tauchi et al. conducted a longitudinal observational study that followed PT infants during their time spent in the NICU.^55^ Results from this study showed that there was a positive association between the abundance of *Bifidobacteriaceae* and the infant transition from an incubator to an open bed.^55^ On the other hand, La Rosa et al. performed a similar analysis where they concluded that there was no association between the gut microbiota composition of infants that were housed in a single room or an open room with multiple subjects.^48^

**Table 6.** Environmental factors and gut microbiota composition of PT infants

Factor	Ref	Alpha diversity	Beta diversity	Taxonomy
***NICU environment***	^89^			Overlap in colonization with *Staphylococcus* and *Enterococcus faecalis* between gut microbiota of PT infant and NICU surfaces
	^90^			Overlap in colonization with *E. faecalis, Staphylococcus epidermis, Klebsiella pneumoniane, Propionibacterium avidu, Escherichia coli* and *Pseudomonas aeruginosa* between gut microbiota of PT infant and NICU surfaces Clostridia found in PT infant, and rarely found in NICU rooms
	^48^			No association between NICU environment (single vs open rooms) and PT infant gut microbiota composition
	^55^			Positive association between gut colonization in PT infants with *Bifidobacteriaceae* and transition from incubator to open bed

NICU: neonatal intensive care unit; PT: preterm.

Two studies conducted by Brooks and collaborators compared the characteristics of the gut microbiota of PT infants with the room environment at the NICU.^89,90^ These studies consisted of collecting stool samples from the infants as wells as medical equipment and surface samples from the NICU room they were housed. These surface samples consisted of the most frequently touched surfaces in the NICU: medical equipment, floors, sinks, computer equipment, counters, coolers, ceilings, and cell phones. An overlap between specific bacteria strains present in the infant’s gut and the NICU surfaces was found, specifically for *Staphylococcus* and E*nterococcus faecalis*.^89^ When they analyzed specific items, they found that the tubing system had the highest abundance of bacteria colonizing the infant’s gut, and the electronics had the lowest abundance.^89^ The other study conducted by the same group, found similar results. Up to twelve bacterial species were shared between the microbiota of the infant’s gut and NICU surfaces.^90^ The species that were more common to overlap between the NICU surfaces and the infant’s gut were *Enterococcus faecalis, Staphylococcus epidermis, Klebsiella pneumoniae, Propionibacterium avidu, Escherichia coli, Pseudomonas aeruginosa*, and to a lesser extent *Staphylococcus aureus, Serratia marcescens, Rothia mucilaginosa, Citrobacter freundii, Streptococcus agalactieae* and *Prevotella bivia*.^90^ Interestingly, although Clostridia is a common colonizer of PT infants gut, this bacteria was rarely found in the NICU room surfaces.^90^

## Discussion

**Figure 2.**
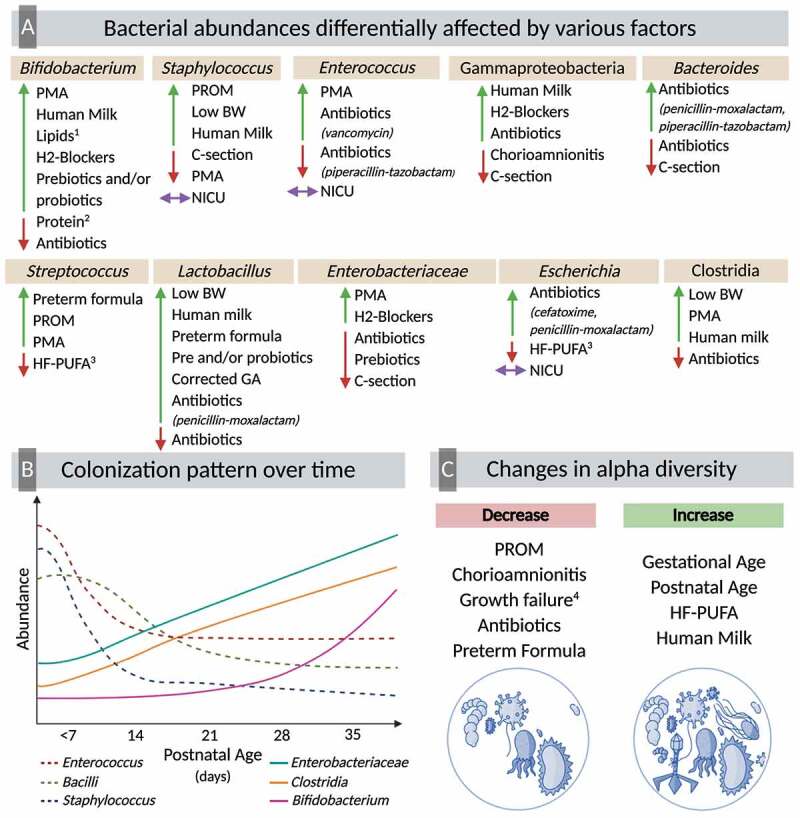
Multifactorial colonization of the preterm gut

The goal of this review was to investigate the perinatal, physiological, dietary, pharmacological, and environmental factors that influence the establishment of the gut bacterial communities in PT infants. A total of 60 publications met the inclusion criteria, reporting changes in alpha diversity, beta diversity, and taxonomic composition of the gut microbiota in response to the various physiological and environmental parameters experienced by PT infants (Figure 2). Nutritional inputs (milk and fortifiers) constituted the largest component of the evidence base.

This figure highlights changes in gut microbiota of PT infants associated to perinatal, physiological, pharmacological, dietary, and environmental factors. A. Based on the literature review, ten bacteria with the most evidence of change across all factors were: *Bifidobacterium, Staphylococcus, Enterococcus*, Gammaproteobacteria, *Bacteroides, Streptococcus, Lactobacillus, Enterobacteriaceae, Escherichia*, and Clostridia. Green arrows denote increase in abundance, red arrows denote decrease in abundance, and purple arrows denote co-colonization between PT gut and that specific factor. B. Bacterial colonization pattern of the gut microbiota of PT infants by postnatal age. After birth, the main colonizers are bacteria from the genera *Enterococcus* (red), *Staphylococcus* (blue), and Bacilli (green). During the first days of life, the abundance of *Enterococcus* and *Staphylococcus* decreases abruptly. With an increase in postnatal age, *Enterobacteriaceae* (teal), *Clostridia* (orange), and *Bifidobacterium* (pink) become more abundant, although the colonization with the latter is delayed in PT infants. Dashed lines represent decrease in abundance. Continuous lines represent increase in abundance. C. Factors affecting alpha diversity. Evidence showed that PROM, chorioamnionitis, growth failure, the exposure to antibiotics and consumption of PT formula decrease alpha diversity. In contrast, gestational age, postnatal age, HF-PUFA enteral supplementation, and human milk (particularly MOM) consumption increase alpha diversity in the gut microbiota of PT infants.^1^ Ratio of grams of lipids to total enteral calories (g/kcal).^2^ Ratio of grams of protein to total enteral calories (g/kcal).^3^ Enteral supplementation.^4^ Growth failure: defined as weight below the 3^rd^ percentile on the Fenton growth charts. BW: birth weight; HF-PUFA: high fat polyunsaturated; MOM: mother’s own milk; NICU: neonatal intensive care unit; PMA: postmenstrual age; PROM: premature rupture of membranes; PT: preterm. Created with BioRender.com with images by MAL.

Modifications in the gut microbiota of PT infants could begin during pregnancy and delivery. The prevailing paradigm in obstetrics has been the sterile womb hypothesis. However, studies have identified the presence of bacteria in the amniotic fluid, placenta, umbilical cord,^91,92^ and meconium of PT,^93^ and full-term infants.^94^ This suggests that colonization of the gastrointestinal tract begins *in utero*.^95^ However, several groups have brought into question whether the detected microbes represent microbial contamination.^96,97^ Two recent studies using microbial culture, qPCR, and DNA sequencing found a lack of evidence for microbes in placental or fetal tissue of rhesus monkeys^98^ or mice.^99^ Nonetheless, PT infants are often exposed to pregnancy-related complications, such as PROM and chorioamnionitis, which can induce PT delivery. These complications were associated with decreased diversity and increased abundance of Gammaproteobacteria, *Staphylococcus, Streptococcus, Serratia*, and *Parabacteroides*.^32,33,35^

In full-term infants, significant differences in fecal microbiota have been reported depending on the mode of delivery,^100^ and these modifications can persist up to one year postpartum.^101^ Many studies in this review reported differences in the structure of the gut microbiota of PT infants depending on delivery mode. Although there was marked variability in the findings related to alpha diversity and beta-diversity, taxonomically, there was a more consistent trend. Vaginal delivery was consistently associated with the presence of *Bacteroides*.^44,49–51^ This observation is in accordance with previous reports showing that full-term infants born via C-section have low *Bacteroides* abundance.^102^ This increase in abundance of *Bacteroides* in vaginally-delivered infants might be attributed to maternal’s fecal microbiota rather that the vaginal microbiota.^103^ The lack of consistency in the results could attributed to the high prevalence of C-section deliveries in PT infants (31%-64%).^6,7^ Thus, further research is needed to clarify the results.

Different physiological factors were explored in this review, including ethnicity, sex, weight, and age. Few studies reported the effect of genetic factors like ethnicity and sex affecting the gut microbiota of PT infants.^33–35,42^ There was not a significant trend that could be drawn from these results. Although previous literature has reported possible differences in the gut microbiota associated with sex^104^ and ethnicity,^105^ insufficient data exists in newborns. There is a strong relationship between PT birth and low birth weight which can affect the fecal microbiota composition. Low birth weight appeared to be associated with higher abundances of Lactobacillales and Clostridiales.^57^ However, these results could be in part explained by the feeding regimen these infants were exposed to, since these findings came from PT infants fed PT formula. In fact, evidence has shown that there are higher abundances of *Clostridium* and *Lactobacillus* in full-term infants fed formula.^106^ In addition to low birth weight, postnatal growth failure is a common feature in PT infants. A study reported the association between diversity, microbiota maturity and growth failure.^58^ A low microbiota-for-age Z-score was found to be prevalent in PT infants.^58^ In children, the microbiota-for-age metric has been linked to modifications in the taxonomical composition related to malnutrition.^107^

In PT infants, both GA at birth and postnatal age are associated with modifications in the structure of the gastrointestinal microbiota. Throughout the literature review, there was consistent evidence of an increase in diversity with greater GA at birth and postnatal age. The most notable changes over time were a decrease in *Enterococcus*, Bacilli and *Staphylococcus* and an increase in *Enterobacteriaceae*, Clostridia and *Bifidobacterium*. It is important to highlight that the colonization with *Bifidobacterium* appears to be delayed in PT infants compared to full-term infants.^55^ Studies in full-term infants have shown that the gut bacterial communities are characterized by low diversity after birth which increases over time and is influenced by dietary factors such as breastfeeding and weaning.^108^ Immediately after birth, the primary gut colonizers are facultative anaerobic bacteria, which reduce the oxygen content of the gut to allow for the subsequent colonization with obligate anaerobes.^109^ *Clostridium* is a strict anaerobe, and some of its members, particularly those from *Clostridium* cluster 1, are associated with prematurity and NEC.^110^ In contrast, the late acquisition of *Bifidobacterium* in PT infants, another strict anaerobe, could be attributed to the lower exposure to human milk compared to full-term infants. The colonization with *Bifidobacterium* has been significantly associated with breastfeeding and human milk consumption in newborns.^111^

The strong relationship between diet and fecal microbiota composition is a well-known fact. Human milk is the gold standard for infant nutrition and plays an essential role in the gut bacterial colonization. The review of the literature showed that in PT infants, this is not an exception. Overall, the consumption of human milk, particularly MOM was associated with greater presence of *Bifidobacterium*^33,68,78^ and *Staphylococcus*.^68,78^ This association could be explained in part to the microbiota composition of human milk. Studies have found that the microbiota composition of human milk is rich primarily in *Staphylococcus*^112,113^ and *Bifidobacterium*.^112^ Nonetheless, there was no consistency across studies between types of milk (human milk [MOM or DHM] or PT formula) and taxonomic composition of the fecal microbiota of PT infants. This could be explained, in part, by the variety of feeding strategies PT infants are exposed to during the hospitalization period. Rates of breastfeeding are lower in PT infants compared to full-term infants;^114^ mothers that deliver prematurely may have little or no milk production caused by immaturity in the mammary gland, illness, or stress.^114^ In the case that PT infants do not receive their MOM, they will be fed DHM or PT formula. Differences in the composition of DHM and PT formula could result in very different gastrointestinal colonization patterns in PT infants. Furthermore, if PT infants are fed MOM or DHM, this will be supplemented with a milk fortifier (human milk-based or bovine milk-based) to achieve adequate nutritional composition and meet the newborn’s needs.^115^ These fortifiers likely further alter gut microbiota composition. With these diverse feeding possibilities, significant and consistent changes will be less likely to be found. For instance, although *Bifidobacterium* is associated with human milk consumption, one study reported a decrease of this bacteria when PT infants were exposed to MOM fortified with a bovine milk fortifier.^28^ This underlines the importance of studies analyzing the gut microbiota taking into account and reporting detailed information regarding the infant’s diet. Additionally, the majority of studies from this literature review were related to dietary factors focused on human milk and/or fortifiers, and very few considered the effect of macronutrients.^64,76^ Results from two studies showed that the protein content, lipid content,^64^ and lipid supplementation^76^ of the diet are associated with the fecal microbiota of PT infants, but more well controlled RCT are needed to further explore these conclusions.

Several studies from this review reported changes in the gut microbiota with the consumption of prebiotics and probiotics, whether used separately or in combination. The most commonly prebiotics administrated were FOS and GOS, and the most common probiotics were *B. breve, B. longum, L. acidophilus*, and *L. rhamnosus*. Most of the significant modifications in the gut microbiota of PT infants were observed with the use of probiotics. As expected, there was a noticeable increase in *Bifidobacterium* and *Lactobacillus* in the infant’s gut with the use of probiotics. Interestingly, the use of probiotics together with human milk (particularly MOM) had an additive effect in increasing *Bifidobacterium* abundance.^30^ Human milk is rich in HMOs,^116^ which are indigestible carbohydrates that are utilized by members of the genera *Bifidobacterium*.^117^ This could explain the differences between PT formula-fed and PT infants exposed to MOM. The use of prebiotics and/or probiotics has shown to decrease colic episodes, decrease fecal pH, improve feeding tolerance and gastric motility, and reduce the risk of allergies.^118^ Extensive research has shown the beneficial effect of probiotics therapy in the reduction of NEC and death in infants born preterm.^119^ However, evidence is still lacking regarding the short- and long-term effects that these probiotics have in the fecal microbiota in PT infants.

The use of antibiotics and the effect they have on the gut microbiota was widely reported across the literature. During hospitalization in the NICU, PT infants are exposed to a variety of medications and antibiotics. It has been reported that up to 89% of preterm infants received antibiotics after birth.^120^ Across the literature, studies found modifications in the diversity and the taxonomical composition of the gut microbiota populations in PT infants after the exposure to antibiotics. Even though antibiotics are prescribed to reduce the number of pathogenic bacteria, the literature review showed this comes accompanied by a decrease in beneficial commensal bacteria like *Bifidobacterium*. The shifts in the overall structure of the gut microbiota are important for the host’s health in the sense that they could cause perturbations in the innate and adaptive immune system.^121^ This is something particularly significant for infants in a fragile state such as PT infants. Moreover, shifts in the gut microbiota appeared to be temporal. This goes in accordance with previous reports showing the transient modifications in the gut microbiota caused by antibiotics exposure, albeit the alteration in the immune system can still occur.^122^ Furthermore, antibiotic-associated alterations in the gut microbiota seem to be dependent on the type of antibiotic. One study reported opposite effects in the abundance of *Enterococcus*; this bacteria decreased with aminoglycoside and increased with vancomycin.^38^ Although vancomycin is used to treat gastrointestinal infections, vancomycin-resistant *Enterococcus* is common nowadays and can be the cause of serious infections in older population.^123^ Antibiotic-resistant bacteria infections have become a public health concern, and data have shown that infants can be colonized with antibiotic-resistant bacteria early in life.^124^ This colonization with antibiotic-resistant bacteria could be coming from environmental, dietary, or maternal factors.^124^ Further exploration of these associations should be conducted to understand the shifts in the bacteria communities of the PT gut. This review also aimed to describe the effect of all commonly administered medications on the gut microbiota composition of PT infants. However, only reports on antibiotics and H2-blockers were found and included in the results. Since PT infants are routinely exposed to a variety of medications during their stay in the NICU, which may modulate the microbiota composition and/or function, additional research that investigates the impact of these other medications is warranted.

Finally, we considered the relationship that exists between the living environment and the gut microbiota. Although the evidence is scarce, two different studies demonstrated associations between the housing environment (incubator or bed, and single vs. open rooms) with the structure of the gut microbiota of PT infants.^48,55^ Only one study found an effect between the infant transition from an incubator to an open bed and colonization with *Bifidobacteriaceae*.^55^ Two different studies, reported how the same bacteria strains were colonizing both the PT gut and many surfaces from the NICU.^89,90^ These similarities between housing environment and the microbiota from different parts of the human body have been previously studied. It has been hypothesized that humans might serve as vectors among multiple room surfaces, and thus, the colonization can be bi-directional.^125^ Hospital-acquired infections are strongly associated with the diversity of microorganisms found in this environment.^126^ This is particularly relevant for PT infants, since these infants are more likely to spend extended periods of time in the NICU. In this environment, a variety of surfaces could serve as sources of microorganisms, including incubators, ventilators, warmers, electronic equipment, as well as health care providers.^22^

This review considers the multifactorial colonization of the PT gut, however, there are some limitations worth mentioning. One limitation of this review is the heterogeneity in the methods for the assessment and analysis of the gut microbiota across studies. As shown **Supplementary Table 1**, the most common method used was NGS (47 studies); from these studies, three performed whole genome sequencing, whereas the rest used 16S rRNA sequencing. Other studies used bacterial culture, molecular methods and other non-sequencing methods such as qPCR, DGGE, TGGE, T-RFLP, PFGE, FISH, MALDI-TOF, and microarrays. The main limitation of bacterial culture and non-sequencing methods is an imprecise characterization of the microbiota diversity.^127^ Also, studies that utilize these methods usually target specific bacteria to answer specific questions, rather than assessing the gut microbiota in a broader way.^127,128^ From the studies that used NGS, there were also differences on the platforms used: pyrosequencing, Illumina dye sequencing or pH-mediated sequencing. The main differences between these techniques are related to read length, reads per run, and reads retained after filtering; where platforms like Illumina will yield more reads and longer reads than the other two platforms.^129^ Thus, this variation in gut microbiota assessment could create biases in the results that have been reported in this review.

Lastly, the available literature regarding the gut microbiota of PT infants relied predominantly on observational studies with very few clinical trials, suggesting the need for more intervention RCTs with adequate power and sample size calculations. After birth, PT infants are at a fragile stage and require a variety of medical interventions. The exposure to different dietary and pharmacological factors will depend on the health status of the infant; this makes it challenging to conduct RCTs in this population. From the review of the literature, almost all the studies of prebiotics and/or probiotics supplementation were clinical trials. These microbiome modulating strategies have been widely used in PT infants and recently, the European Society for Pediatric Gastroenterology Hepatology and Nutrition (ESPGHAN) recommended additional RCT to study the effect of probiotics in infants born preterm.^130^

## Conclusions and future directions

Results from this literature review about the multifactorial colonization in the PT infant gut highlights how multiple factors and different exposures can differently modify the abundance or presence of bacteria from the same genera or class, as shown in (Figure 2). Although changes in numerous bacteria were found across perinatal, physiological, dietary, pharmacological, and environmental factors, some bacteria consistently showed differences across the mentioned factors. These bacteria included *Bifidobacterium, Staphylococcus, Enterococcus*, Gammaproteobacteria, *Bacteroides, Streptococcus, Lactobacillus, Enterobacteriaceae, Escherichia and* Clostridia. The results of this systematic review also illustrate the variability in some of the associations that have been reported with the gut microbiota, which highlights the need of more comprehensive studies analyzing the effect of mode of delivery, sex, type of milk consumed, use of fortifiers, and use of medications on the composition of the gut microbiota of PT infants. Infants born preterm most likely will be affected by multiple conditions at the same time including C-section delivery, antibiotics exposure, low birth weight, and different feeding regimes. With the rapid advancement in sequencing technologies, such as long-read 16S rRNA sequencing that allow for a deeper resolution of the gut microbiome, coupled with the use of more sophisticated computational tools, future biomedical research should aim to integrate multiple biological inputs, seeking to understand complex systems such as the gut microbiota of PT infants. From a systems biology perspective, this would encompass studying the associations between bacterial genome, infant’s metabolome, immune markers, clinical status, dietary factors, and the effect on the infant health outcomes. Robust associations support the need for prospective RCTs to utilize modifiable factors, such as diet, to mitigate the adverse effects of non-modifiable factors, including low GA or low birth weight, to help prevent or ameliorate detrimental complications associated with the common dysbiosis associated with PT birth.

## Supplementary Material

Supplemental Material
