# Supplementary material for: A systematic review of the factors influencing microbial colonization of the preterm infant gut
Source: Gut Microbes. 2021 Apr 4;13(1):1884514. doi: 10.1080/19490976.2021.1884514 (PMC8023245; doi:10.1080/19490976.2021.1884514)
Supplement: Supplemental Material [file KGMI_A_1884514_SM1633.docx]

**Supplemental Table 1. Gut microbiota characterization tools.**

| **Author** | **Gut microbiota assessment method** | **DNA extraction** | **16S rRNA region** | **Sequencing platform** | **Data analysis pipeline** | **Reference database** |
| --- | --- | --- | --- | --- | --- | --- |
| Adbulkadir, et al. ^1^ | NGS  qPCR (*Lactobacillus acidophilus* and *Bifidobacterium bifidum*) | PowerSoil DNA Isolation Kit  MoBio | V4 | Illumina MiSeq | Morthur | RDP |
| Aly, et al. ^2^ | Bacterial Culture  qPCR (*B. bifidum* and *Lactobacillus spp*) | QIAamp DNA Stool Mini Kit  QIAGEN | NA | NA | NA | NA |
| Arboleya, et al. ^3^ | NGS  qPCR | QIAamp DNA Stool Mini Kit  QIAGEN | V3-V4 | Ion Torrent | QIIME | RDP |
| Armanian, et al ^4^ | Bacterial Culture | NA | NA | NA | NA | NA |
| Biagi, et al. ^5^ | NGS | DNeasy Blood & Tissue Kit  QIAGEN | V3-V4 | Illumina MiSeq | QIIME | RDP |
| Brooks, et al. ^6^ | NGS | PowerSoil DNA Isolation Kit  MoBio | Full length | Illumina  HiSeq | USEARCH | Silva |
| Brooks, et al. ^7^ | NGS | PowerSoil DNA Isolation Kit  MoBio | WGS | Illumina  HiSeq | Bowtie2  Idba_ud  USEARCH | NS |
| Brown, et al. ^8^ | NGS | PowerSoil DNA Isolation Kit  MoBio | WGS | Illumina  HiSeq | Bowtie2  Idba_ud  USEARCH | NS |
| Butcher, et al. ^9^ | NGS | FastDNA Spin Kit  MP Biomedicals | V6 | Illumina  HiSeq | QIIME | Greengenes |
| Cai, et al. ^10^ | NGS | Fecal DNA MiniPrepTM Kit  Zymo Research | V3-V4 | Illumina  MiSeq | QIIME | Greengenes |
| Chernikova, et al. ^11^ | NGS | PowerSoil DNA Isolation Kit  MoBio | V4-V6 | 454 Life Sciences GS FLX | GAST | SILVA |
| Chernikova, et al. ^12^ | NGS | PowerSoil DNA Isolation Kit  MoBio  Fecal DNA MiniPrepTM Kit  Zymo Research | V4-V5 | Illumina  MiSeq | GAST | SILVA |
| Cong, et al. ^13^ | NGS | PowerSoil DNA Isolation Kit  MoBio | V4 | Illumina  MiSeq | QIIME | Greengenes |
| Costello, et al. ^14^ | NGS | QIAamp DNA Stool Mini Kit  QIAGEN | V3-V5 | 454 Life Sciences GS FLX | QIIME | RDP |
| Dahl, et al. ^15^ | NGS | Mechanical lysis with beads | V4 | Illumina  HiSeq | QIIME | Greengenes |
| Esaiassen, et al. ^16^ | NGS | NorDiag Arrow Stool Dna Extraction kit  NorDiag | V3 | Illumina  MiSeq | MetaPhlan | NS |
| Forsgren, et al. ^17^ | qPCR (*Bifidobacterium, Bifidobacterium adolescentis, B. bifidum, Bifidobacterium breve, Bifidobacterium catenulatum, Bifidobacterium lactis, Bifidobacterium longum, Bifidobacterium infantis, Clostridium coccoides, Clostridium leptum, Clostridium difficile, Clostridium perfringens, Staphylococcus aureus* and *Akkermancia muciniphila*) | InviMag Stool DNA kit  Stratec Molecular | NA | NA | NA | NA |
| Gibson, et al. ^18^ | NGS | PowerMax Soil DNA Isolation kit  MoBio | V4 | Illumina  MiSeq | MetaPhlan | NS |
| Gómez, et al. ^19^ | NGS | Mechanical lysis with beads and phenol-chloroform extraction | T7 promoter-carrying gene | HITChip | Agilent Feature Extraction | NS |
| Gregory, et al. ^20^ | qPCR (*Bifidobacterium fragilis*) | Stool DNA extraction kit  QIAGEN | NA | NA | NA | NA |
| Gregory, et al. ^21^ | NGS | Stool DNA extraction kit  MoBio | V4-V5 | Illumina  MiSeq | QIIME | RDP |
| Grier, et al. ^22^ | NGS | Fecal DNA kit  QIAGEN | V3-V4 | Illumina  MiSeq | QIIME | Greengenes |
| Gupta, et al. ^23^ | NGS | QIAamp DNA Stool Mini Kit  QIAGEN | V1-V3 | 454 Life Sciences GS FLX | QIIME | RDP |
| Ho, et al. ^24^ | NGS | PowerSoil DNA Isolation Kit  MoBio | V4 | Illumina  MiSeq | CLC Biomedical Workbench | Greengenes |
| Ishizeki, et al. ^25^ | Bacterial Culture | NA | NA | NA | NA | NA |
| Korpela, et al. ^26^ | NGS | Repeated bead beating method | V1-V3 | Illumina  MiSeq | USEARCH | NS |
| La Rosa, et al. ^27^ | NGS | Standardized protocols from HPMC | V3-V5 | 454 Life Sciences GS FLX | Naive Bayesian Classifier | RDP |
| Mai, et al. ^28^ | DGGE  qPCR (*Bifidobacterium*)  NGS | Stool DNA extraction kit  QIAGEN | V6-V8 | 454 Life Sciences GS FLX | ESPRIT  QIIME | NS |
| Millar, et al. ^29^ | NGS | QIAamp DNA Stool Mini Kit  QIAGEN | V1-V3 | 454 Life Sciences GS FLX | Morthur | RDP |
| Moles, et al. ^30^ | Bacterial Culture  DGGE  NGS | Mechanical lysis with beads and phenol-chloroform extraction | V1-V6 | HITChip | Agilent Feature Extraction Software | NA |
| Moles, et al. ^31^ | Bacterial Culture  MALDI-TOF  PFGE | NS | NA | NA | NA | NA |
| Mshvildadze, et al. ^32^ | DGGE  NGS | Stool DNA extraction kit  QIAGEN | V6-V8 | 454 Life Sciences GS FLX | RDP pipeline | RDP |
| Normann, et al. ^33^ | NGS | UltraClean fecal DNA Isolation Kit  MoBio | V3-V4 | 454 Life Sciences GS FLX | RDP pipeline | RDP |
| Parra-Llorca, et al. ^34^ | NGS | MAsterPure Complete DNA & RNA Purification Kit  Epicentre | V3-V4 | Illumina  MiSeq | QIIME | Greengenes |
| Pärtty, et al. ^35^ | FISH  qPCR (*Bifidobacterium, Bacteroides-Prevotella, Clostridium histolytucum, Lactobacillus-Enterococcus* and *A. muciniphila*) | InviMag Stool DNA Kit  Stratec Molecular | NA | NA | NA | NA |
| Patel, et al. ^36^ | qPCR (*Bifidobacterium* spp)  NGS | FastDNA Spin Kit for soil  MP Biomedicals | Full length | 454 Life Sciences GS FLX | QIIME | Greengenes |
| Poroyko, et al. ^37^ | NGS | QIAamp DNA Stool Mini Kit  QIAGEN | V1-V4 | 454 Life Sciences GS FLX | RDP pipeline | RDP |
| Ravi, et al. ^38^ | NGS | QIAamp DNA Stool Mini Kit  QIAGEN | V3-V4 | Illumina MiSeq | QIIME | Greengenes |
| Rougé, et al. ^39^ | Bacterial Culture  TGGE | NA | NA | NA | BIBI  Blast  Multalin  ClustalW | NA |
| Rozé, et al. ^40^ | Bacterial Culture  NGS | Protocol SOP07  IHMS | V3-V4 | 454 Life Sciences GS FLX | NS | NS |
| Sherman, et al. ^41^ | NGS | QIAamp DNA Stool Mini Kit  QIAGEN | V1-V3 | 454 Life Sciences GS FLX | QIIME  Morthur | NS |
| Sim, et al. ^42^ | NGS | FastDNA Spin Kit for soil  MP Biomedicals | V3-V5 | 454 Life Sciences GS FLX | QIIME | RDP |
| Soeorg, et al. ^43^ | Bacterial Culture | NA | NA | NA | NA | NA |
| Stewart, et al. ^44^ | NGS | PowerSoil DNA Isolation Kit  MoBio | V4 | Illumina  MiSeq | USEARCH | Silva |
| Tauchi, et al. ^45^ | NGS | Mechanical lysis with beads and phenol-chloroform extraction | V1-V2 | Illumina  MiSeq | QIIME | Living Tree Database |
| Underwood, et al. ^46^ | TRFLP (Bacilli specific)  qPCR (*Bifidobacterium*) | QIAamp DNA Stool Mini Kit  QIAopfgpGEN | NA | NA | QIIME | RDP |
| Underwood, et al. ^47^ | NGS | ZR-96 Kit  SPEX SamplePrep | V4 | Illumina  MiSeq | QIIME | Greengenes |
| Underwood, et al. ^48^ | NGS | ZR-96 Kit  SPEX SamplePrep | V4 | Illumina  MiSeq | QIIME | Greengenes |
| Underwood, et al. ^49^ | TRFLP (Bacilli and *Bifidobacterium* specific  qPCR (Eubacteria and *Bifidobacterium*) | QIAamp DNA Stool Mini Kit  QIAGEN | NA | NA | QIIME | RDP |
| Underwood, et al. ^50^ | Bacterial Culture  qPCR | Details on request to the author | NA | NA | NA | NA |
| Wandro, et al. ^51^ | NGS  qPCR | Fecal DNA MiniPrepTM Kit  Zymo Research | V3-V4 | Illumina  MiSeq | QIIME | Greengenes |
| Westerbeek, et al. ^52^ | FISH | NA | NA | NA | NA | NA |
| Younge, et al. ^53^ | NGS | Soil Microbe DNA Kit  Zymo Research | V4 | Illumina  HiSeq | QIIME | Silva |
| Younge, et al. ^54^ | NGS | Fecal DNA MiniPrepTM Kit  Zymo Research | V4 | Illumina MiSeq | QIIME | Silva |
| Zeber-Lubecka, et al. ^55^ | NGS | QIAamp DNA Stool Kit  QIAGEN | WGS | Ion Torrent | Morthur | Silva |
| Zhou, et al. ^56^ | NGS | QIAamp DNA Stool Mini Kit  QIAGEN | V3-V5 | 454 Life Sciences GS FLX | HMP protocol | RDP |
| Zhu, et al. ^57^ | NGS | QIAamp DNA Stool Mini Kit  QIAGEN | V3-V4 | Illumina  MiSeq | QIIME | RDP |
| Zou, et al. ^58^ | NGS | QIAamp DNA Stool Mini Kit  QIAGEN | V3-V4 | Illumina  MiSeq | NS | NS |
| Zwittink, et al. ^59^ | NGS  qPCR (*Bifidobacterium*, *Enterococcus* and *Enterobacteriaceae*) | Mechanical lysis with beads and phenol-chloroform extraction | V3-V4 | 454 Life Sciences GS FLX | QIIME | Silva |
| Zwittink, et al. ^60^ | NGS | Mechanical lysis with beads and phenol-chloroform extraction | V3-V5 | 454 Life Sciences GS FLX | QIIME | Silva |

DGGE: denaturing gradient gel electrophoresis; FISH: fluorescent in situ hybridization; HMP: human microbiome project; HPMC: Human Pan-Microbe Communities; IHMS: International Human Microbiome Standards; MALDI-TOF: matrix assisted laser desorption/ionization time-of-flight mass spectrometry; NA: not applicable; NGS: next generation sequencing; NS: not specified; PFGE: pulsed-field gel electrophoresis; qPCR: quantitative polymerase chain reaction; RDP: ribosomal database project; TGGE: temperature gradient gel electrophoresis; TRFLP: terminal restriction fragment length polymorphism; WGS: whole genome sequencing.

**Supplemental Table 2. Risk of bias scores based on the RoB2* tool for clinical trials.**

| **Author** | **Risk of bias from the randomization process** | **Risk of bias due to deviation from the intended interventions** | **Missing data** | **Risk of bias in measurement of the outcomes** | **Risk of bias in selection of the reported results** | **Overall risk of bias** |
| --- | --- | --- | --- | --- | --- | --- |
| Adbulkadir, et al. ^1^ | Some concerns | Low | Low | Low | Low | Some concerns |
| Aly, et al. ^2^ | Low | Low | Low | Low | Low | Low |
| Armanian, et al ^4^ | Low | Low | Low | Low | Low | Low |
| Ishizeki, et al. ^25^ | Low | Low | Low | Low | Low | Low |
| Millar, et al. ^29^ | Low | Low | Low | Low | Low | Low |
| Pärtty, et al. ^35^ | Low | Low | Low | Low | Low | Low |
| Rougé, et al. ^39^ | Low | Low | Low | Low | Low | Low |
| Sherman, et al. ^41^ | Low | Low | Some concerns | High | High | High |
| Underwood, et al. ^46^ | Low | Some concerns | Low | Low | Low | Some concerns |
| Underwood, et al. ^47^ | Low | Some concerns | Low | Low | Low | Some concerns |
| Underwood, et al. ^48^ | Low | Some concerns | Low | Low | Low | Some concerns |
| Underwood, et al. ^50^ | Low | Low | Low | Low | Low | Low |
| Westerbeek, et al. ^52^ | Low | Low | Low | Low | Low | Low |
| Younge, et al. ^53^ | Low | Low | Low | Low | Low | Low |

* RoB2 assesses risk of bias based on five different domains providing an overall score. In each section, three possible scores are possible: low risk of bias, some concerns or high risk of bias.

**Supplemental Table 3. Quality of the included studies by the Newcastle-Ottawa scale for cohort studies*.**

| **Author** | **Selection** | | | | **Comparability** | **Outcomes** | | |
| --- | --- | --- | --- | --- | --- | --- | --- | --- |
|  | *Representativeness of the exposed cohort* | *Selection of the non-exposed cohort* | *Ascertainment of exposure* | *Demonstration that outcomes of interest was not present at start of study* | *Comparability of cohort on the bases of the design or analysis* | *Assessment of outcomes* | *Was follow-up long enough for outcomes to occur* | *Adequacy of follow-up of cohort* |
| Biagi, et al. ^5^ | * | NA | * | * | NA | * | * | * |
| Brooks, et al. ^6^ | * | NA | * | * | NA | * | * | * |
| Brooks, et al. ^7^ | * | NA | * | * | NA | * | * | * |
| Brown, et al. ^8^ | * | NA | * | * | NA | * | * | * |
| Butcher, et al. ^9^ | * | NA | * | * | NA | * | * | * |
| Cai, et al. ^10^ | * | * | * | * | ** | * | * | * |
| Chernikova, et al. ^11^ | * | NA | * | * | NA | * | * | * |
| Chernikova, et al. ^12^ | * | * | * | * | ** | * | * | * |
| Cong, et al. ^13^ | * | NA | * | * | NA | * | * | * |
| Costello, et al. ^14^ | * | NA | * | * | NA | * | * | * |
| Dahl, et al. ^15^ | * | * | * | * | ** | * | * | * |
| Forsgren, et al. ^17^ | * | * | * | * | ** | * | * | * |
| Gibson, et al. ^18^ | * | * | * | * | ** | * | * | * |
| Gómez, et al. ^19^ | * | NA | * | * | NA | * | * | * |
| Gregory, et al. ^20^ | * | NA | * | * | NA | * | * | * |
| Gregory, et al. ^21^ | * | * | * | * | ** | * | * | * |
| Grier, et al. ^22^ | * | NA | * | * | NA | * | * | * |
| Ho, et al. ^24^ | * | NA | * | * | NA | * | * | * |
| Korpela, et al. ^26^ | * | NA | * | * | NA | * | * | * |
| La Rosa, et al. ^27^ | * | NA | * | * | NA | * | * | * |
| Moles, et al. ^30^ | * | NA | * | * | NA | * | * | * |
| Moles, et al. ^31^ | * | NA | * | * | NA | * | * | * |
| Mshvildadze, et al. ^32^ | * | * | * | * | ** | * | * | * |
| Parra-Llorca, et al. ^34^ | * | * | * | * | ** | * | * | * |
| Patel, et al. ^36^ | * | NA | * | * | NA | * | * | * |
| Ravi, et al. ^38^ | * | * | * | * | ** | * | * | * |
| Rozé, et al. ^40^ | * | NA | * | * | ** | * | * | * |
| Soeorg, et al. ^43^ | * | * | * | * | ** | * | * | * |
| Stewart, et al. ^44^ | * | * | * | * | ** | * | * | * |
| Tauchi, et al. ^45^ | * | * | * | * | ** | * | * | * |
| Underwood, et al. ^49^ | * | NA | * | * | NA | * | * | * |
| Wandro, et al. ^51^ | * | NA | * | * | NA | * | * | * |
| Younge, et al. ^54^ | * | * | * | * | ** | * | * | * |
| Zwittink, et al. ^59^ | * | NA | * | * | NA | * | * | * |

* The Newcastle-Ottawa Scale covers four domains (participant selection, comparability, exposure and outcomes), measured by eight different questions. One star is given to each question, except for the comparability section that can receive one or two stars. Longitudinal studies can score up to 9 stars. Studies that did not have a non-exposed group were scored with NA (not applicable) in two sections.

**Supplemental Table 4. Quality of the included studies by the Newcastle-Ottawa scale for case-control studies*.**

| **Author** | **Selection** | | | | **Comparability** | **Exposure** | | |
| --- | --- | --- | --- | --- | --- | --- | --- | --- |
|  | *Is the case definition adequate* | *Representativeness of the cases* | *Selection of controls* | *Definition of controls* | *Comparability of cases and controls on the basis of the design or analysis* | *Ascertainment of exposure* | *Same method of ascertainment for cases and controls* | *Non-response rate* |
| Arboleya, et al. ^3^ | * | * | * | * | ** | * | * | * |
| Esaiassen, et al. ^16^ | * | * | * | * | ** | * | * | * |
| Gupta, et al. ^23^ | * | * | * | * | ** | * | * | * |
| Mai, et al. ^28^ | * | * | * | * | ** | * | * | * |
| Normann, et al. ^33^ | * | * | * | * | ** | * | * | * |
| Poroyko, et al. ^37^ | * | * | * | * | ** | * | * | * |
| Sim, et al. ^42^ | * | * | * | * | ** | * | * | * |
| Zhou, et al. ^56^ | * | * | * | * | ** | * | * | * |
| Zhu, et al. ^57^ | * | * | * | * | ** | * | * | * |
| Zou, et al. ^58^ | * | * | * | * | ** | * | * | * |
| Zwittink, et al. ^60^ | * | * | * | * | ** | * | * | * |

* The Newcastle-Ottawa Scale covers four domains (participant selection, comparability, exposure and outcomes), measured by eight different questions. One star is given to each question, except for the comparability section that can receive one or two stars. Case-control studies can score up to 9 stars.

# References

1. Abdulkadir B, Nelson A, Skeath T, Marrs ECL, Perry JD, Cummings SP, Embleton ND, Berrington JE, Stewart CJ. Routine Use of Probiotics in Preterm Infants: Longitudinal Impact on the Microbiome and Metabolome. Neonatology 2016; 109:239–47.

2. Aly H, Said RN, Wali IE, Elwakkad A, Soliman Y, Awad AR, Shawky MA, Alam MSA, Mohamed MA. Medically Graded Honey Supplementation Formula to Preterm Infants as a Prebiotic. J Pediatr Gastroenterol Nutr 2017; 64:966–70.

3. Arboleya S, Sánchez B, Milani C, Duranti S, Solís G, Fernández N, de los Reyes-Gavilán CG, Ventura M, Margolles A, Gueimonde M. Intestinal Microbiota Development in Preterm Neonates and Effect of Perinatal Antibiotics. J Pediatr 2015; 166:538–44.

4. Armanian A-M, Sadeghnia A, Hoseinzadeh M, Mirlohi M, Feizi A, Salehimehr N, Torkan M, Shirani Z. The effect of neutral oligosaccharides on fecal microbiota in premature infants fed exclusively with breast milk: A randomized clinical trial. J Res Pharm Pract 2016; 5:27.

5. Biagi E, Aceti A, Quercia S, Beghetti I, Rampelli S, Turroni S, Soverini M, Zambrini AV, Faldella G, Candela M, et al. Microbial Community Dynamics in Mother’s Milk and Infant’s Mouth and Gut in Moderately Preterm Infants. Front Microbiol 2018; 9:2512.

6. Brooks B, Firek BA, Miller CS, Sharon I, Thomas BC, Baker R, Morowitz MJ, Banfield JF. Microbes in the neonatal intensive care unit resemble those found in the gut of premature infants. Microbiome 2014; 2:1.

7. Brooks B, Olm MR, Firek BA, Baker R, Thomas BC, Morowitz MJ, Banfield JF. Strain-resolved analysis of hospital rooms and infants reveals overlap between the human and room microbiome. Nat Commun 2017; 8:1814.

8. Brown CT, Xiong W, Olm MR, Thomas BC, Baker R, Firek B, Morowitz MJ, Hettich RL, Banfield JF. Hospitalized Premature Infants Are Colonized by Related Bacterial Strains with Distinct Proteomic Profiles. MBio 2018; 9.

9. Butcher J, Unger S, Li J, Bando N, Romain G, Francis J, Mottawea W, Mack D, Stintzi A, O’Connor DL. Independent of Birth Mode or Gestational Age, Very-Low-Birth-Weight Infants Fed Their Mothers’ Milk Rapidly Develop Personalized Microbiotas Low in Bifidobacterium. J Nutr 2018; 148:326–35.

10. Cai C, Zhang Z, Morales M, Wang Y, Khafipour E, Friel J. Feeding practice influences gut microbiome composition in very low birth weight preterm infants and the association with oxidative stress: A prospective cohort study. Free Radic Biol Med 2019; 142:146–54.

11. Chernikova DA, Koestler DC, Hoen AG, Housman ML, Hibberd PL, Moore JH, Morrison HG, Sogin ML, Zain-ul-abideen M, Madan JC. Fetal exposures and perinatal influences on the stool microbiota of premature infants. J Matern Neonatal Med 2016; 29:99–105.

12. Chernikova DA, Madan JC, Housman ML, Zain-ul-abideen M, Lundgren SN, Morrison HG, Sogin ML, Williams SM, Moore JH, Karagas MR, et al. The premature infant gut microbiome during the first 6 weeks of life differs based on gestational maturity at birth. Pediatr Res 2018; 84:71–9.

13. Cong X, Judge M, Xu W, Diallo A, Janton S, Brownell EA, Maas K, Graf J. Influence of Feeding Type on Gut Microbiome Development in Hospitalized Preterm Infants. Nurs Res 2017; 66:123–33.

14. Costello EK, Carlisle EM, Bik EM, Morowitz MJ, Relman DA. Microbiome Assembly across Multiple Body Sites in Low-Birthweight Infants. MBio 2013; 4.

15. Dahl C, Stigum H, Valeur J, Iszatt N, Lenters V, Peddada S, Bjørnholt J V, Midtvedt T, Mandal S, Eggesbø M. Preterm infants have distinct microbiomes not explained by mode of delivery, breastfeeding duration or antibiotic exposure. Int J Epidemiol 2018; 47:1658–69.

16. Esaiassen E, Hjerde E, Cavanagh JP, Pedersen T, Andresen JH, Rettedal SI, Støen R, Nakstad B, Willassen NP, Klingenberg C. Effects of Probiotic Supplementation on the Gut Microbiota and Antibiotic Resistome Development in Preterm Infants. Front Pediatr 2018; 6.

17. Forsgren M, Isolauri E, Salminen S, Rautava S. Late preterm birth has direct and indirect effects on infant gut microbiota development during the first six months of life. Acta Paediatr 2017; 106:1103–9.

18. Gibson MK, Wang B, Ahmadi S, Burnham C-AD, Tarr PI, Warner BB, Dantas G. Developmental dynamics of the preterm infant gut microbiota and antibiotic resistome. Nat Microbiol 2016; 1:16024.

19. Gómez M, Moles L, Espinosa-Martos I, Bustos G, de Vos W, Fernández L, Rodríguez J, Fuentes S, Jiménez E. Bacteriological and Immunological Profiling of Meconium and Fecal Samples from Preterm Infants: A Two-Year Follow-Up Study. Nutrients 2017; 9:1293.

20. Gregory KE, LaPlante RD, Shan G, Kumar DV, Gregas M. Mode of Birth Influences Preterm Infant Intestinal Colonization With Bacteroides Over the Early Neonatal Period. Adv Neonatal Care 2015; 15:386–93.

21. Gregory KE, Samuel BS, Houghteling P, Shan G, Ausubel FM, Sadreyev RI, Walker WA. Influence of maternal breast milk ingestion on acquisition of the intestinal microbiome in preterm infants. Microbiome 2016; 4:68.

22. Grier A, Qiu X, Bandyopadhyay S, Holden-Wiltse J, Kessler HA, Gill AL, Hamilton B, Huyck H, Misra S, Mariani TJ, et al. Impact of prematurity and nutrition on the developing gut microbiome and preterm infant growth. Microbiome 2017; 5:158.

23. Gupta RW, Tran L, Norori J, Ferris MJ, Eren AM, Taylor CM, Dowd SE, Penn D. Histamine-2 Receptor Blockers Alter the Fecal Microbiota in Premature Infants. J Pediatr Gastroenterol Nutr 2013; 56:397–400.

24. Ho TTB, Groer MW, Kane B, Yee AL, Torres BA, Gilbert JA, Maheshwari A. Dichotomous development of the gut microbiome in preterm infants. Microbiome 2018; 6:157.

25. Ishizeki S, Sugita M, Takata M, Yaeshima T. Effect of administration of bifidobacteria on intestinal microbiota in low-birth-weight infants and transition of administered bifidobacteria: A comparison between one-species and three-species administration. Anaerobe 2013; 23:38–44.

26. Korpela K, Blakstad EW, Moltu SJ, Strømmen K, Nakstad B, Rønnestad AE, Brække K, Iversen PO, Drevon CA, de Vos W. Intestinal microbiota development and gestational age in preterm neonates. Sci Rep 2018; 8:2453.

27. La Rosa PS, Warner BB, Zhou Y, Weinstock GM, Sodergren E, Hall-Moore CM, Stevens HJ, Bennett WE, Shaikh N, Linneman LA, et al. Patterned progression of bacterial populations in the premature infant gut. Proc Natl Acad Sci 2014; 111:12522–7.

28. Mai V, Torrazza RM, Ukhanova M, Wang X, Sun Y, Li N, Shuster J, Sharma R, Hudak ML, Neu J. Distortions in Development of Intestinal Microbiota Associated with Late Onset Sepsis in Preterm Infants. PLoS One 2013; 8:e52876.

29. Millar M, Seale J, Greenland M, Hardy P, Juszczak E, Wilks M, Panton N, Costeloe K, Wade WG. The Microbiome of Infants Recruited to a Randomised Placebo-controlled Probiotic Trial (PiPS Trial). EBioMedicine 2017; 20:255–62.

30. Moles L, Gómez M, Heilig H, Bustos G, Fuentes S, de Vos W, Fernández L, Rodríguez JM, Jiménez E. Bacterial Diversity in Meconium of Preterm Neonates and Evolution of Their Fecal Microbiota during the First Month of Life. PLoS One 2013; 8:e66986.

31. Moles L, Gómez M, Jiménez E, Fernández L, Bustos G, Chaves F, Cantón R, Rodríguez JM, del Campo R. Preterm infant gut colonization in the neonatal ICU and complete restoration 2 years later. Clin Microbiol Infect 2015; 21:936.e1-936.e10.

32. Mshvildadze M, Neu J, Shuster J, Theriaque D, Li N, Mai V. Intestinal Microbial Ecology in Premature Infants Assessed with Non–Culture-Based Techniques. J Pediatr 2010; 156:20–5.

33. Normann E, Fahlén A, Engstrand L, Lilja HE. Intestinal microbial profiles in extremely preterm infants with and without necrotizing enterocolitis. Acta Paediatr 2013; 102:129–36.

34. Parra-Llorca A, Gormaz M, Alcántara C, Cernada M, Nuñez-Ramiro A, Vento M, Collado MC. Preterm Gut Microbiome Depending on Feeding Type: Significance of Donor Human Milk. Front Microbiol 2018; 9:1376.

35. Pärtty A, Luoto R, Kalliomäki M, Salminen S, Isolauri E. Effects of Early Prebiotic and Probiotic Supplementation on Development of Gut Microbiota and Fussing and Crying in Preterm Infants: A Randomized, Double-Blind, Placebo-Controlled Trial. J Pediatr 2013; 163:1272-1277.e2.

36. Patel AL, Mutlu EA, Sun Y, Koenig L, Green S, Jakubowicz A, Mryan J, Engen P, Fogg L, Chen AL, et al. Longitudinal Survey of Microbiota in Hospitalized Preterm Very-Low-Birth-Weight Infants. J Pediatr Gastroenterol Nutr 2016; 62:292–303.

37. Poroyko V, Morowitz M, Bell T, Ulanov A, Wang M, Donovan S, Bao N, Gu S, Hong L, Alverdy JC, et al. Diet creates metabolic niches in the “inmature gut” that shape microbial communities. Nutr Hosp 2011; 26:1283–95.

38. Ravi A, Estensmo ELF, Abée-Lund TML, Foley SL, Allgaier B, Martin CR, Claud EC, Rudi K. Association of the gut microbiota mobilome with hospital location and birth weight in preterm infants. Pediatr Res 2017; 82:829–38.

39. Rougé C, Piloquet H, Butel M-J, Berger B, Rochat F, Ferraris L, Des Robert C, Legrand A, de la Cochetière M-F, N’Guyen J-M, et al. Oral supplementation with probiotics in very-low-birth-weight preterm infants: a randomized, double-blind, placebo-controlled trial. Am J Clin Nutr 2009; 89:1828–35.

40. Rozé J-C, Ancel P-Y, Lepage P, Martin-Marchand L, Al Nabhani Z, Delannoy J, Picaud J-C, Lapillonne A, Aires J, Durox M, et al. Nutritional strategies and gut microbiota composition as risk factors for necrotizing enterocolitis in very-preterm infants. Am J Clin Nutr 2017; 106:821–30.

41. Sherman MP, Sherman J, Arcinue R, Niklas V. Randomized Control Trial of Human Recombinant Lactoferrin: A Substudy Reveals Effects on the Fecal Microbiome of Very Low Birth Weight Infants. J Pediatr 2016; 173:S37–42.

42. Sim K, Shaw AG, Randell P, Cox MJ, McClure ZE, Li M-S, Haddad M, Langford PR, Cookson WOCM, Moffatt MF, et al. Dysbiosis Anticipating Necrotizing Enterocolitis in Very Premature Infants. Clin Infect Dis 2015; 60:389–97.

43. Soeorg H, Metsvaht T, Eelmäe I, Merila M, Treumuth S, Huik K, Jürna-Ellam M, Ilmoja M-L, Lutsar I. The role of breast milk in the colonization of neonatal gut and skin with coagulase-negative staphylococci. Pediatr Res 2017; 82:759–67.

44. Stewart CJ, Embleton ND, Clements E, Luna PN, Smith DP, Fofanova TY, Nelson A, Taylor G, Orr CH, Petrosino JF, et al. Cesarean or Vaginal Birth Does Not Impact the Longitudinal Development of the Gut Microbiome in a Cohort of Exclusively Preterm Infants. Front Microbiol 2017; 8:1008.

45. Tauchi H, Yahagi K, Yamauchi T, Hara T, Yamaoka R, Tsukuda N, Watanabe Y, Tajima S, Ochi F, Iwata H, et al. Gut microbiota development of preterm infants hospitalised in intensive care units. Benef Microbes 2019; 10:641–51.

46. Underwood MA, Salzman NH, Bennett SH, Barman M, Mills DA, Marcobal A, Tancredi DJ, Bevins CL, Sherman MP. A Randomized Placebo-controlled Comparison of 2 Prebiotic/Probiotic Combinations in Preterm Infants: Impact on Weight Gain, Intestinal Microbiota, and Fecal Short-chain Fatty Acids. J Pediatr Gastroenterol Nutr 2009; 48:216–25.

47. Underwood MA, Kalanetra KM, Bokulich NA, Lewis ZT, Mirmiran M, Tancredi DJ, Mills DA. A Comparison of Two Probiotic Strains of Bifidobacteria in Premature Infants. J Pediatr 2013; 163:1585-1591.e9.

48. Underwood MA, Kalanetra KM, Bokulich NA, Mirmiran M, Barile D, Tancredi DJ, German JB, Lebrilla CB, Mills DA. Prebiotic Oligosaccharides in Premature Infants. J Pediatr Gastroenterol Nutr 2014; 58:352–60.

49. Underwood MA, Gaerlan S, De Leoz MLA, Dimapasoc L, Kalanetra KM, Lemay DG, German JB, Mills DA, Lebrilla CB. Human milk oligosaccharides in premature infants: absorption, excretion, and influence on the intestinal microbiota. Pediatr Res 2015; 78:670–7.

50. Underwood MA, Davis JCC, Kalanetra KM, Gehlot S, Patole S, Tancredi DJ, Mills DA, Lebrilla CB, Simmer K. Digestion of Human Milk Oligosaccharides by Bifidobacterium breve in the Premature Infant. J Pediatr Gastroenterol Nutr 2017; 65:449–55.

51. Wandro S, Osborne S, Enriquez C, Bixby C, Arrieta A, Whiteson K. The Microbiome and Metabolome of Preterm Infant Stool Are Personalized and Not Driven by Health Outcomes, Including Necrotizing Enterocolitis and Late-Onset Sepsis. mSphere 2018; 3.

52. Westerbeek EAM, Slump RA, Lafeber HN, Knol J, Georgi G, Fetter WPF, Elburg RM. The effect of enteral supplementation of specific neutral and acidic oligosaccharides on the faecal microbiota and intestinal microenvironment in preterm infants. Eur J Clin Microbiol Infect Dis 2013; 32:269–76.

53. Younge N, Yang Q, Seed PC. Enteral High Fat-Polyunsaturated Fatty Acid Blend Alters the Pathogen Composition of the Intestinal Microbiome in Premature Infants with an Enterostomy. J Pediatr 2017; 181:93-101.e6.

54. Younge NE, Newgard CB, Cotten CM, Goldberg RN, Muehlbauer MJ, Bain JR, Stevens RD, O’Connell TM, Rawls JF, Seed PC, et al. Disrupted Maturation of the Microbiota and Metabolome among Extremely Preterm Infants with Postnatal Growth Failure. Sci Rep 2019; 9:8167.

55. Zeber-Lubecka N, Kulecka M, Ambrozkiewicz F, Paziewska A, Lechowicz M, Konopka E, Majewska U, Borszewska-Kornacka M, Mikula M, Cukrowska B, et al. Effect of Saccharomyces boulardii and Mode of Delivery on the Early Development of the Gut Microbial Community in Preterm Infants. PLoS One 2016; 11:e0150306.

56. Zhou Y, Shan G, Sodergren E, Weinstock G, Walker WA, Gregory KE. Longitudinal Analysis of the Premature Infant Intestinal Microbiome Prior to Necrotizing Enterocolitis: A Case-Control Study. PLoS One 2015; 10:e0118632.

57. Zhu D, Xiao S, Yu J, Ai Q, He Y, Cheng C, Zhang Y, Pan Y. Effects of One-Week Empirical Antibiotic Therapy on the Early Development of Gut Microbiota and Metabolites in Preterm Infants. Sci Rep 2017; 7:8025.

58. Zou Z-H, Liu D, Li H-D, Zhu D-P, He Y, Hou T, Yu J-L. Prenatal and postnatal antibiotic exposure influences the gut microbiota of preterm infants in neonatal intensive care units. Ann Clin Microbiol Antimicrob 2018; 17:9.

59. Zwittink RD, van Zoeren-Grobben D, Martin R, van Lingen RA, Groot Jebbink LJ, Boeren S, Renes IB, van Elburg RM, Belzer C, Knol J. Metaproteomics reveals functional differences in intestinal microbiota development of preterm infants. Mol Cell Proteomics 2017; 16:1610–20.

60. Zwittink RD, Renes IB, van Lingen RA, van Zoeren-Grobben D, Konstanti P, Norbruis OF, Martin R, Groot Jebbink LJM, Knol J, Belzer C. Association between duration of intravenous antibiotic administration and early-life microbiota development in late-preterm infants. Eur J Clin Microbiol Infect Dis 2018; 37:475–83.
